# The pivotal role of astrocytes in an *in vitro* stroke model of the blood-brain barrier

**DOI:** 10.3389/fncel.2014.00352

**Published:** 2014-10-28

**Authors:** Winfried Neuhaus, Fabian Gaiser, Anne Mahringer, Jonas Franz, Christoph Riethmüller, Carola Förster

**Affiliations:** ^1^Department of Pharmaceutical Chemistry, University of ViennaVienna, Austria; ^2^Department of Anesthesia and Critical Care, University Hospital WürzburgWürzburg, Germany; ^3^Department of Pharmaceutical Technology and Biopharmacy, Institute of Pharmacy and Molecular Biotechnology, University of HeidelbergHeidelberg, Germany; ^4^Serend-ip GmbH, Centre for NanotechnologyMünster, Germany

**Keywords:** blood-brain barrier, *in vitro*, stroke, oxygen/glucose deprivation, ischemia, traumatic brain injury, cerebEND, C6

## Abstract

Stabilization of the blood-brain barrier during and after stroke can lead to less adverse outcome. For elucidation of underlying mechanisms and development of novel therapeutic strategies validated *in vitro* disease models of the blood-brain barrier could be very helpful. To mimic *in vitro* stroke conditions we have established a blood-brain barrier *in vitro* model based on mouse cell line cerebEND and applied oxygen/glucose deprivation (OGD). The role of astrocytes in this disease model was investigated by using cell line C6. Transwell studies pointed out that addition of astrocytes during OGD increased the barrier damage significantly in comparison to the endothelial monoculture shown by changes of transendothelial electrical resistance as well as fluorescein permeability data. Analysis on mRNA and protein levels by qPCR, western blotting and immunofluorescence microscopy of tight junction molecules claudin-3,-5,-12, occludin and ZO-1 revealed that their regulation and localisation is associated with the functional barrier breakdown. Furthermore, soluble factors of astrocytes, OGD and their combination were able to induce changes of functionality and expression of ABC-transporters Abcb1a (P-gp), Abcg2 (bcrp), and Abcc4 (mrp4). Moreover, the expression of proteases (matrixmetalloproteinases MMP-2, MMP-3, MMP-9, and t-PA) as well as of their endogenous inhibitors (TIMP-1, TIMP-3, PAI-1) was altered by astrocyte factors and OGD which resulted in significant changes of total MMP and t-PA activity. Morphological rearrangements induced by OGD and treatment with astrocyte factors were confirmed at a nanometer scale using atomic force microscopy. In conclusion, astrocytes play a major role in blood-brain barrier breakdown during OGD *in vitro*.

## Introduction

Impairment of the blood-brain barrier (BBB) leads to vasogenic edema and subsequent brain damage after acute cerebral ischemic insults such as stroke or traumatic brain injury. Reduced permeability of the BBB is associated with decreased harm and improved outcome after stroke (Yang and Rosenberg, 2011). Consequently, elucidation of underlying mechanisms causing BBB breakdown may lead to novel targets for future treatments to reduce adverse stroke outcomes. The BBB itself is considered as the most important interface between blood circulation and the central nervous system (CNS). Physiological roles of the BBB are to maintain the homoestasis in the CNS, to prevent the passage of bacteria, viruses and unwelcomed substances into the CNS and to efflux CNS waste products. In the 1960's Reese and Brightman found out by electron microscopy that the brain capillary endothelium is the main component of the BBB, which is sealed by intercellular tight junctions. In addition to the presence of tight junctions further major differences of the brain capillary endothelium in comparison to the peripheral endothelium is the lack of fenestrae and reduced pinocytotic activity (Brightman and Reese, 1969; Joo, 1996). During ischemia several key properties of the BBB are changed. The physical barrier is disrupted which results in increased permeability for marker molecules of the paracellular route such as albumin bound Evans blue, fluorescent labeled dextrans, fluorescein, mannitol, and sucrose. This loss of barrier function against hydrophilic compounds was associated with alterations within the tight junctional complex reflected by decreased expression or delocalisation of tight junction proteins such as occludin (András et al., 2007; Kleinschnitz et al., 2011). Furthermore, changed expression and functionality of ATP-binding cassette (ABC)-transporter proteins such as P-glycoprotein (P-gp or Abcb1) and breast cancer resistance protein (bcrp or Abcg2) were reported under ischemic or hypoxic conditions. One of the consequences would be an altered transport barrier accompanied with a loss of the protective function against a wide array of xenobiotics. In addition, the activity of proteases such as matrixmetalloproteinases (MMPs) and tissue-plasminogen activator (t-PA) is increased during stroke that was shown to be associated with degradation of the basal lamina and integrity loss of the BBB (reviewed in Jin et al., 2010 and Patak and Hermann, 2011).

Nowadays, the BBB is understood as a complex regulated system. Terms such as neuro- or gliavascular unit (NVU, GVU) describe the strong influence of the microenvironment on the brain endothelium. Neighboring cell types such as astrocytes, pericytes, microglia or even neurons are known to influence the functionality of the BBB in health as well as in disease, which is supported by their physical proximity and consequent small diffusion distances for signaling molecules (Abbott et al., 2006; Cecchelli et al., 2007). During ischemia the undersupply with glucose and oxygen causes reduced ATP production and loss of available energy at the NVU. This contributes significantly to the breakdown of the BBB (Ronaldson and Davis, 2012). Therefore, application of oxygen/glucose deprivation (OGD) on CNS cells *in vitro* is an established model for stroke. Recent studies showed that BBB *in vitro* models based on brain endothelial cells co- or even triple-cultured with astrocytes and/or pericytes are able to reflect the physiology of the BBB in a more accurate manner (Török et al., 2003; Nakagawa et al., 2009; Ceruti et al., 2011). In this context, in case of *in vitro* stroke models of the BBB, lately reports confirmed that astrocytes aggravated the breakdown of the physical barrier (Mysiorek et al., 2009). However, underlying mechanisms were not described in detail and the need to investigate and understand them seemed to be essential for validation purposes. In previous studies, we have applied and optimized oxygen/glucose deprivation (OGD) conditions for brain endothelial mono-cultures to mimick stroke *in vitro* and to study molecular mechanisms lying behind the successful, functional stabilization of the BBB *in vivo* during stroke or traumatic brain injury (Kleinschnitz et al., 2011; Neuhaus et al., 2012a; Thal et al., 2013). The aim of the present study was to extend our stroke *in vitro* models of the BBB with astrocytes by co-cultivation of mouse BBB cell line cerebEND with rat cell line C6 and to investigate the influence of astrocytes under OGD-conditions on several BBB relevant parameters such as functionality of the physical as well as transport barrier, associated tight junction molecule and Abc-transporter expression, expression and functionality of MMPs, t-PA and of their endogenous inhibitors, and last but not least whether morphological changes were detectable via atomic force microscopy.

## Materials and methods

### Materials

Collagen IV from human placenta (C5533), PBS (D8537), Triton-X 100 (T8787), DMEM (D5796), Calcein-AM (17783), DAPI (D8417), db-cAMP (D0260), MK571 (M7571), Ko143 (K2144), verapamil.HCl (V4629), β-mercaptoethanol (M6250), fluorescein sodium (F6377), albumin from bovine serum for immunofluorescence microscopy (fraction V, A9647) and for western blotting (A7906) were purchased from Sigma-Aldrich. Bodipy-FL-prazosin (B-7433), DMEM without glucose (11966-025, Gibco®) was obtained from Life technologies (USA), and fluo-cAMP (F002-01) was from Biolog (Bremen, Germany). FCS Gold EU approved was bought from PAA Laboratories (A15151, Lot A15111-2018, Linz, Austria) and was heat-inactivated in a water-bath at 56°C for 30 min. Penicillin/streptomycin (100X, 10,000 Units/mL, 10,000 μg/mL, A2213) and 0.05% Tyrpsin/0.02% EDTA-solution (L2143) were from BioChrom AG (Berlin, Germany). 6-well, 12-well and 24-well plates and 24-well Transwell® inserts (0.4 μm pore size, PET) were obtained from Becton and Dickinson (REF353046, REF353043, REF353095, REF353226, USA). Gelatine was from SERVA (22151, Heidelberg, Germany), nuclease-free water was purchased from Ambion (AM9937, USA). All other substances were of analytical grade.

### Cell culture

Mouse brain endothelial cell line cerebEND was produced from isolated brain microvascular endothelial cells from cerebellum by Silwedel and Förster (2006). cerebENDs were cultured in DMEM medium supplemented with 10% FCS and 1% penicillin/streptomycin in 0.5% gelatine coated cell culture tissue flasks and were subcultivated by trypsination in a ratio of 1:3 once a week as published recently (Neuhaus et al., 2012a). Rat glioma cell line C6 was obtained from ATCC and cultured with the same medium as cerebENDs in 0.5% gelatine coated cell culture tissue flasks. Subcultivation was accomplished in a ratio of 1:20 once a week. Cells were maintained in an incubator at 37°C, 95% humidity and a 5% CO2/95% air atmosphere.

### Transwell experiments

**Figure 1:**
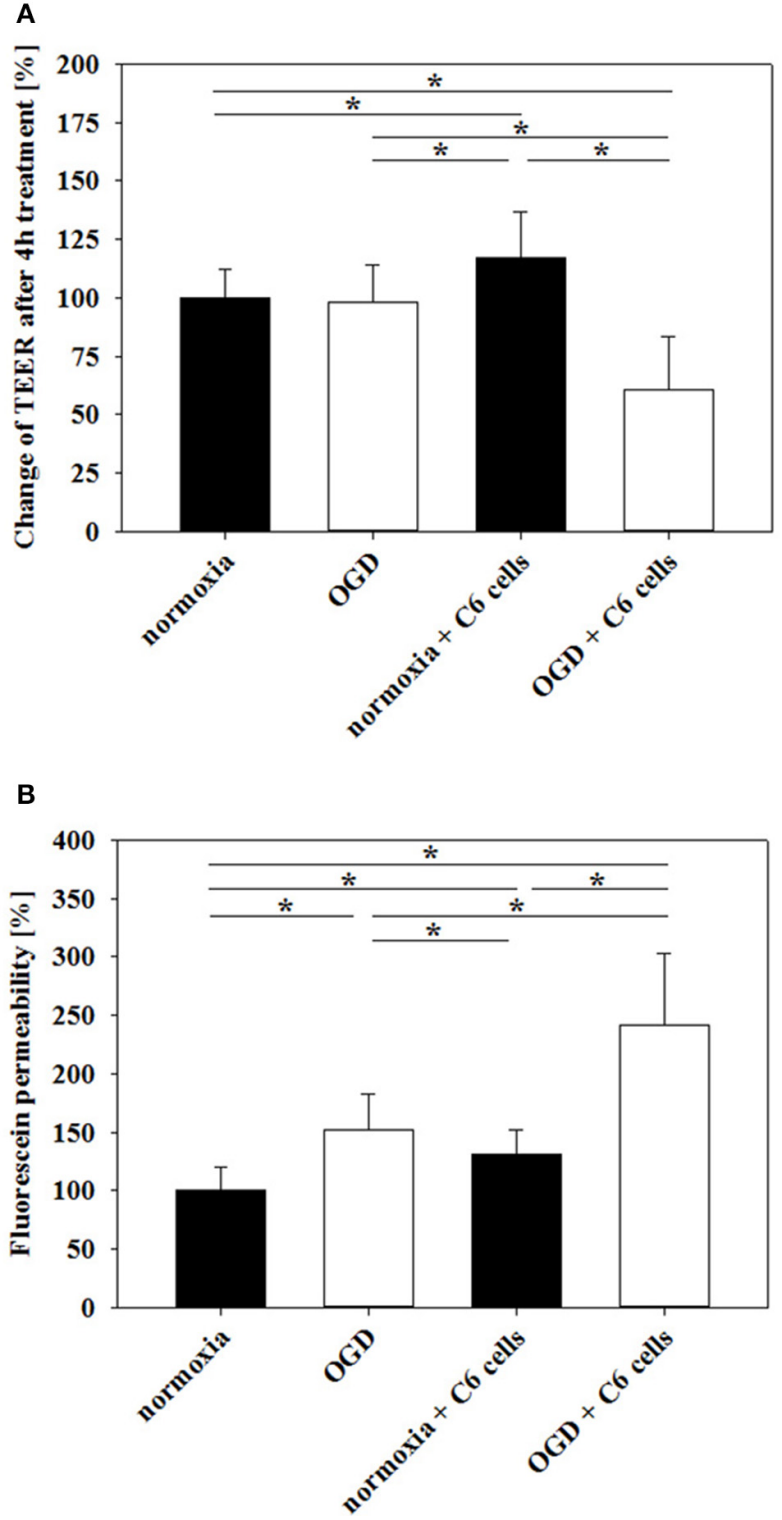
**Influence of 4 h oxygen/glucose deprivation (OGD) treatment on barrier functionality of mono-cultured cerebEND layers or co-cultured with astrocytes (C6) on Transwell inserts measured by TEER (A) or fluorescein permeability (B)**. Statistical significance was labeled with ^*^(*p* < 0.05, two-sided student's *t*-test with same variances). Data are presented as means ± *SD* (*n* = 11–59).

24-well plate inserts were coated with 100 μg/mL collagen IV (dissolved in acetic acid according to the manufacturer's instruction) at RT for 2 h and were washed with PBS for three times to get rid off the residual acetic acid. CerebENDs were seeded at a density of 40,000 cells/cm^2^ on the 24-well inserts, and every other day growth medium was renewed. On day 7 after seeding apical growth medium was supplemented with 100 nM hydrocortisone (100 μM stock dissolved in ethanol) and C6 cells were seeded at a density of 20,000 cells/cm^2^ in 0.5% gelatine coated 24-well plates. On day 9 inserts with cerebENDs were placed over C6 cell containing well-plates to form the co-culture set-up still with 100 nM hydrocortisone in the apical chamber. The Transwell insert cultivation procedure was summarized in Figure 1S. Oxygen/glucose deprivation (OGD) experiments were conducted on day 13. cerebEND cells in inserts and C6 cells in well-plates were washed with PBS twice before they were reesembled and serum-free DMEM with or without glucose was added. Transendothelial electrical resistance (TEER) was measured using chopstick electrodes from Millipore after 30 min of equibrilation at RT as previously published (Neuhaus et al., 2006). To apply OGD, cells in serum-free DMEM without glucose were incubated in a hypoxia incubator (HERACELL 150i, ThermoFisher, USA) with 1% O_2_, 5% CO_2_, saturated humidity atmosphere and 37°C for 4 h. As controls, blank inserts without cells, mono-cultured cerebEND cells and cerebENDs co-cultured with C6 cells were incubated in a normoxia incubator at air O_2_, 5% CO_2_, saturated humidity atmosphere and 37°C for the same time. After incubation TEER was measured again after a 30 min temperature equibrilation phase. To study fluorescein permeability of cell layers, apical experimental medium was exchanged with 300 μL DMEM with or without glucose containing 10 μM fluorescein and transport study was accomplished for 1 h at 37°C under normoxic conditions. DMEM with or without glucose as blank solutions, basolateral samples and residual apical stock solutions were measured in 100 μL triplicates in black 96-well plates (GreinerBioOne, Kremsmünster, Austria) with a fluorescence microplate reader (GeniosPro, Tecan, Austria) at 485/535 nm (excitation/emission wavelength). TEER in Ohm/cm^2^ and permeability coefficients including blank insert values were calculated as previously published (Neuhaus et al., 2008; Novakova et al., 2014). Due to comparison reasons, values of normoxic mono-cultured cerebEND controls were set to 100%.

### Quantitative reverse-transcriptase polymerase chain reaction (qPCR)

For qPCR experiments, cerebEND and C6 cells were seeded in gelatine (0.5%, Serva, Germany) coated 6-well plates at a density of 40,000 cells/cm^2^ or 20,000 cells/cm^2^, respectively. Every other day growth medium was renewed. On day 6 after seeding, cells were washed with PBS twice, and 3 mL of serum-free DMEM with or without glucose was added, and normoxia or OGD treatment was applied for 4 h as described above. Four different kinds of treatments were conducted: N = normoxia, OGD = oxygen/glucose deprivation, N-C6 = normoxia of cerebEND cells with supernatant of normoxic treated C6 cells and OGD-C6 = oxygen/glucose deprivation of cerebEND cells with supernatant of OGD-treated C6 cells. In case of N-C6 and OGD-C6 treatments, C6 cells were incubated for 4 h normoxia or OGD to obtain their supernatants which were directly applied on PBS washed cerebEND cell layers to guarentee fresh soluble factors of C6 cells for further 4 h incubation. After N, N-C6, OGD or OGD-C6 treatment of cerebEND cells experimental media were removed and lysed in RA1 buffer (supplemented with 1% β-mercaptoethanol shortly before usage) of the Nucleospin-RNAII Kit (Macherey Nagel, Düren, Germany). Total RNA was isolated using the Nucleospin-RNAII Kit according to the manufacturer's instruction as described before in Neuhaus et al. (2012b). RNA concentrations were determined by means of a Nanodrop ND 2000 spectrophotometer (FisherScientific, Schwerte, Germany) at 260/280 nm. 1 μg total RNA per sample were reversely transcribed to 20 μL cDNA by means of the high capacity cDNA-kit (with random primer and RNAse inhibitor) from Applied Biosystems (Life Technologies GmbH, Darmstadt, Germany) according to the manufacturer's instruction. qPCR analysis were performed using FAM-labeled probes for all investigated targets (Taqman®, Applied Biosystems) as recently reported (Neuhaus et al., 2012b). A detailed list with the product numbers of Taqman®-probes could be found in the Supplementary part (Table 1S). Each sample was analyzed as triplicate. Relative mRNA abundances to β-actin were calculated by the ddCt method using following formula: 2^(Ct of β-actin-Ct of gene of interest)^, where Ct is the threshold cycle value.

### Western blotting

Cells were cultured as described above under Section Quantitative Reverse-Transcriptase Polymerase Chain Reaction (qPCR) and scraped after the treatments in 50 μL RIPA buffer per 6-well [50 mM TRIS pH 8; 150 mM NaCl, 0.1% SDS, 0.5% sodium-deoxycholate, 1% NP40 supplemented with one complete ULTRA protease inhibitor cocktail and PhosphoSTOP minitablet per 10 mL (complete ULTRA tablets, Mini, EASYpack, REF05892970001; PhosSTOP, REF04906837001, Roche Applied Science, Mannheim, Germany)] on ice after washing with ice-cold PBS twice as described previously (Neuhaus et al., 2012b). In case of membran protein enrichment, proteins were extracted with 1% Triton-X 100 in PBS (supplemented with one complete ULTRA protease inhibitor cocktail and PhosphoSTOP minitablet per 10 mL) gentle shaking at 4°C for 30 min after washing with ice-cold PBS on ice twice. Sample's protein concentrations were determined by a detergent-compatible Pierce BCA assay (FisherScientific) utilizing a BSA standard curve (Albumin Standard, FisherScientific). Before storage at −80°C 4x Laemmli buffer (8% SDS, 40% glycerol, 0.004% bromphenolic blue, 0.25 M Tris-HCl supplemented with 6% β-mercaptoethanol shortly before usage) was added to the samples. 20 μg protein of total RIPA-cell lysates and 15 μg of Triton-X 100 fractions per lane and peqGOLD prestained protein marker V (PEQLAB) were loaded onto 7.5, 10 or 12% SDS-PAGE gels (1.5 mm thick) after ultrasound treatment and 5 min denaturation at 70°C. After gel electrophoresis at 130V, proteins were immunoblotted onto polyvinylidene difluoride membranes (162–0177, Biorad, München, Germany) by a tank blotter at 40 mA per gel at 4°C overnight. Incubations with primary and secondary antibodies were carried out as previously described (Neuhaus et al., 2012b). Used primary and secondary antibodies are listed in the Supplementary Table 2S. To visualize the bands, western blots were incubated with ECL-solutions for 3 min and were developed using a FluorChem FC2 Multiimager II (Alpha Innotech, Hessisch Oldendorf, Germany). Density values of single protein bands were calculated with the software Alpha View and were related to the corresponding β-actin bands. In some cases, antibodies onto western blots were stripped by washing with H_2_O dest. for 5 min followed by a treatment with 0.2 M NaOH for 5 min and a second washing step with H_2_O dest. for 5 min. After an additional washing step with PBS-T, membranes were blocked with 5% milk powder before reprobing with the next primary antibody. In case of strong antibody-protein binding, antibodies were stripped by washing 10 min with PBS-T for three times and incubated with a 100 mM ß-mercaptoethanol containing buffer (62.5 mM Tris-HCl *pH* = 6.8, 2% SDS, freshly added ß-mercaptoethanol) at 56°C and gentle shaking for 20 min followed by three washing steps with PBS-T each for 10 min. Finally, membranes were blocked with 5% milk powder as before.

### Immunofluorescence microscopy

Immunofluorescence microscopy was carried out as published recently (Neuhaus et al., 2008, 2012b). In brief, cerebEND cell layers (cultured as explained above on 15 mm, with ethanol disinfected, PBS washed and collagen IV coated glas slides in 12-well plates) were washed for three times with PBS and fixed and permeabilized with precooled MeOH at −20°C for 20 min. After washing with PBS twice, cells were rehydrated with PBS at RT for 15 min. In case of staining with the mouse anti-occludin antibody, cells were fixed with ice-cold ethanol incubated at −20°C for 20 min followed by −20°C cold acetone at room temperature for 3 min and drying after acetone removal at room temperature for 10 min. Then, cell layers were incubated with primary antibody solutions (see Supplementary Table 2S) at 37°C for 1 h. After washing for three times with PBS cell layers were incubated with 1:100 1% BSA/PBS solutions with the secondary antibodies (see Supplementary Table 2S) at 37°C for 30 min. For nuclei staining an additional incubation step of 10 min at 37°C with 1:3000 of 5 mg/mL DAPI in PBS was carried out. After washing with PBS for three times, cover slips were transferred to glas slides and were embedded in Vectashield Hard-Set mounting medium (Vector Laboratories LTD., Peterborough, United Kingdom). Images were generated by using an Olympus BX51 microscopy system using UPlan FLN objectives (4x/0.13/∞/-/FN26.5; 10x/0.30//∞/-/FN26.5; 20x/0.50/∞/0.17/FN26.5; 40x/0.75/∞/0.17/FN26.5; 60x/0.90/0.11-0.23/FN26.5) equipped with U-RFL-T laser and controlled by the software cellSense Dimension.

### Uptake assays

cerebEND cells were seeded in 200 μL growth medium at a density of 40,000 cells/cm^2^ in clear, gelatine (0.5%) coated 96-well plates (GreinerBioOne, Kremsmünster, Austria). On day 6 after seeding, cells were washed with 200 μL prewarmed HBSS per 96-well twice and incubated in 100 μL appropriate medium for 4 h. For normoxia (N) serum-free DMEM containing glucose, for OGD serum-free DMEM without glucose, for N-C6 sterile filtered, serum-free DMEM with glucose preincubated on washed C6 cell layers over night and for OGD-C6 sterile filtered, serum-free DMEM without glucose preincubated on washed C6 cell layers under OGD conditions over night were used. In case of OGD and OGD-C6 with cerebEND cells, medium was changed after 4 h of incubation in the hypoxia chamber to normoxic or N-C6 medium (i.e., serum-free DMEM with glucose or sterile-filtered, serum-free DMEM with glucose preincubated with C6 cells over night) for further 20 h in the normxia chamber to simulate reoxygenation/renutrition. After 24 h of incubation in total, medium in selected wells was changed with 100 μL appropriate experimental media containing specific ABC-transporter inhibitors [Abcb1: 100 μM verapamil (100 mM stock in DMSO), Abcc4: 10 μM MK571 (10 mM stock in DMSO), 100–500 μM db-cAMP (100 mM stock in H_2_O), Abcg2: 5 μM Ko143 (5 mM stock in DMSO)] for 15–30 min preincubation at 37°C. After that, uptake of specific substrates (end concentrations: 1 μM calcein-AM for Abcb1, 10 μM fluo-cAMP for Abcc4 (Reichel et al., 2010), 0.5 μM Bodipy-FL-prazosin for Abcg2) was started by addition of 100 μL of appropriate experimental media containing 2-fold substrate concentrations and in case additionally one-fold inhibitor concentration. Stock solutions of substrates (1 mM Calcein-AM in DMSO, 2 mM fluo-cAMP in H_2_O, 500 μM Bodipy-FL-prazosin in DMSO) were thawed in darkness shortly before addition to ice-cold experimental media prepared in darkness to prevent degradation of light-sensitive substrates. After 45 (Abcb1), 90 (Abcc4) or 120 (Abcg2) minutes of incubation at 37°C, cells were washed on ice with 200 μL/well ice-cold HBSS for three times and lysed with 1% Triton-X 100 solution (in HBSS) for at least 1 h during gently shaking in darkness. Then, fluorescence of uptaken substrates was measured at 485/535 nm excitation/emission wavelength by means of a fluorescence microplate reader (GeniosPro, Tecan, Austria), substracted by background fluorescence of cells and related to their protein concentration determined by BCA method as described above in Section Western Blotting. Control wells after 24 h of normoxia were set to 100% and effects of added inhibitors under each condition were calculated. Changes of efflux functionality were presented as differences in inhibitor dependent uptake percentages in order to minimize the influence of other unkown, possibly regulated transporters on the total uptake of the used substrates. Prelimenary tests with used transporter inhibitors verapamil (Abcb1), MK571 (Abcc4) and Ko143 (Abcg2) confirmed their specificity for investigated transporters in cerebEND cells (see Supplementary Figure 2S).

### Measurement of enzyme activities

To measure enzyme activity of matrixmetalloproteinases (MMP) or of tissue plasminogen activator (t-PA), medium supernatants of cerebENDs after N, N-C6, OGD or OGD-C6 treatments or C6 cells after N or OGD treatment were collected and stored at −80°C until measurement. 6 mL medium pooled from two six-wells were concentrated to 200 μL by centrifugation at 4000 g and 4°C for 40 min using 10 kDa Amicon ultrafiltration tubes (UFC901024, Millipore, Germany). In case of MMP activity, 80 μL of retentates were mixed with 50 μL of 1:10 diluted MMP substrate with assay buffer (520 MMP FRET substrate SB-14, 100 μM stock dissolved in 10% DMSO in assay buffer, 60581-01, Anaspec, USA) in a black 96-well microplate (GreinerBioOne, Kremsmünster, Austria). The MMP substrate is sensitive to detect activity of MMP-1, -2, -3, -7, -8, -9, -12, and -13. Increasing fluorescence was recorded over 120 min at 37°C and 485/535 nm in a microplate reader (GeniosPro, Tecan, Austria). Control blank values with pure DMEM were substracted from MMPs containing media and slopes between 10 and 60 min were calculated after linear regression analysis. Values were corrected for minor deviations of obtained retentat volumina after the centrifugation before. Slopes of media supernatants of cerebEND cells treated for 4 h of normoxia (N) were set to 100%. In case of t-PA activity, 80 μL of retentats were mixed with 50 μL of 1:100 diluted t-PA substrate with assay buffer (component A of Sensolyte AMC t-PA activity assay kit, 72160, Anaspec, USA) in a black 96-well microplate and fluorescence was recorded over 120 min at 37°C and 360/460 nm in a microplate reader. Control blank values with pure DMEM were substracted from t-PA containing media and slopes between 10 and 120 min were calculated after linear regression analysis. Values were corrected for minor deviations of obtained retentat volumina after the centrifugation before. Slopes of media supernatants of cerebEND cells treated for 4 h of normoxia (N) were set to 100%.

### Surface nano-texture analysis (nAnostic)

Contact mode Atomic force microscopy (AFM) on cultivated cells was performed as described before (Jungmann et al., 2008). In this study, cells were chemically stabilized by glutardialdehyde fixation (1% final concentration). Briefly, AFM measurements were carried out in PBS-buffered solution (pH 7.4) using a Multimode AFM equipped with Nanoscope III controller and software version 5.30sr3 (Digital Instruments, Santa Barbara, CA, USA). Silicon-nitride tips on V-shaped gold-coated cantilevers were used (0.01 N/m, MLCT, VEECO, Mannheim, Germany). Imaging was performed at ambient temperature with forces less than 1 nN at 1–3 scan lines per second (1–3 Hz) with 512^*^512 pixels resolution. For texture analysis, subcellular scan areas of (20 μm)^2^ are recorded. Topographical data of the cell surfaces were analyzed using the nAnostic™-method applying custom-built, proprietary algorithms (Serend-ip GmbH, Münster, Germany). The method principle has been described before (Thoelking et al., 2010). Briefly, each nanostructure protruding from the mean surface level is morphometrically evaluated. Then, they are filtered by their size and shape through computer vision; here, only structures of positive local deviational volume (LDV), with LDV less than 1 cubed μm (10^3^ nm height, 10^3^nm^*^10^3^nm area) were considered. The average volume of thousands of objects analyzed per sample was 0.0021 μm^3^. Values are given for the count of such objects (per 20 μm image). Ten (20 μm)^2^ areas per slide were arbitrarily chosen and evaluated from two independent experiments. To comply with inbuilt biological heterogeneity the number of analyzed areas was set to *n* = 20 for statistical significance.

## Results

### Astrocytes increase OGD-induced damage of the blood-brain barrier

Based on preliminary experiments and literature data of OGD-treated murine brain endothelial cells (Kleinschnitz et al., 2011), it was decided to concentrate on 4 h OGD-treatments with 1% O_2_ in glucose-, and serum-free medium. Prior studies showed that cerebENDs reacted on 4 h OGD-treatment with significant upregulation of HIF1α (1.5-fold) and VEGFa (11-14-fold) (Supplementary Figure 3S, further targets angiopoietin-2, caveolin-1, Lrp-1, neuropilin-1 and Tie-2 see Supplementary Table 3S), while cell viability remained unaffected (Neuhaus et al., 2012a). Differences of electrical resistance raw data between cell and blank inserts in serum-free media before OGD-treatment were in average about 80–82 Ω for mono-cultured and 62–63 Ω for cerebENDs co-cultured with C6 cells resulting in calculated TEER values in average of 250 Ω /cm^2^ or 200 Ω /cm^2^, respectively. Measuring changes of TEER before and after OGD-treatment revealed no significant decrease of TEER of mono-cultured cerebEND layers [100 ± 12% normoxia (*n* = 42) vs. 98 ± 16% OGD (*n* = 11)] (Figure 1A). On the contrary, comparison of cerebENDs co-cultivated with C6 cells showed a significant increase in TEER to 117 ± 20% (*p* < 0.05, *n* = 24) after 4 h serum-free normoxia and a significant decrease to 61 ± 23% (*p* < 0.05, *n* = 59) after 4 h OGD treatment. Data of fluorescein permeability studies under normoxic conditions after TEER measurement—equivalent with a 1 h reoxygenation phase—confirmed the deleterious effects of C6 cells on tightness of cerebEND cell layers during OGD, but also uncovered harmful effects of OGD on mono-cultured cerebENDs (Figure 1B). In detail, fluorescein permeability was significantly increased by OGD to 152 ± 31% (*p* < 0.05, *n* = 11) in comparison to normoxic controls (100 ± 20%, *n* = 31). In case of C6 co-cultered cerebENDs, fluorescein permeability was 131 ± 21% (*n* = 18) under normoxic conditions in comparison to the normoxic mono-cultured cerebEND layers and was significantly elevated to 242 ± 61% by OGD treatment (*p* < 0.05, *n* = 50).

### Influence of astrocytes and OGD on expression of tight junction proteins

**Figure 2:**
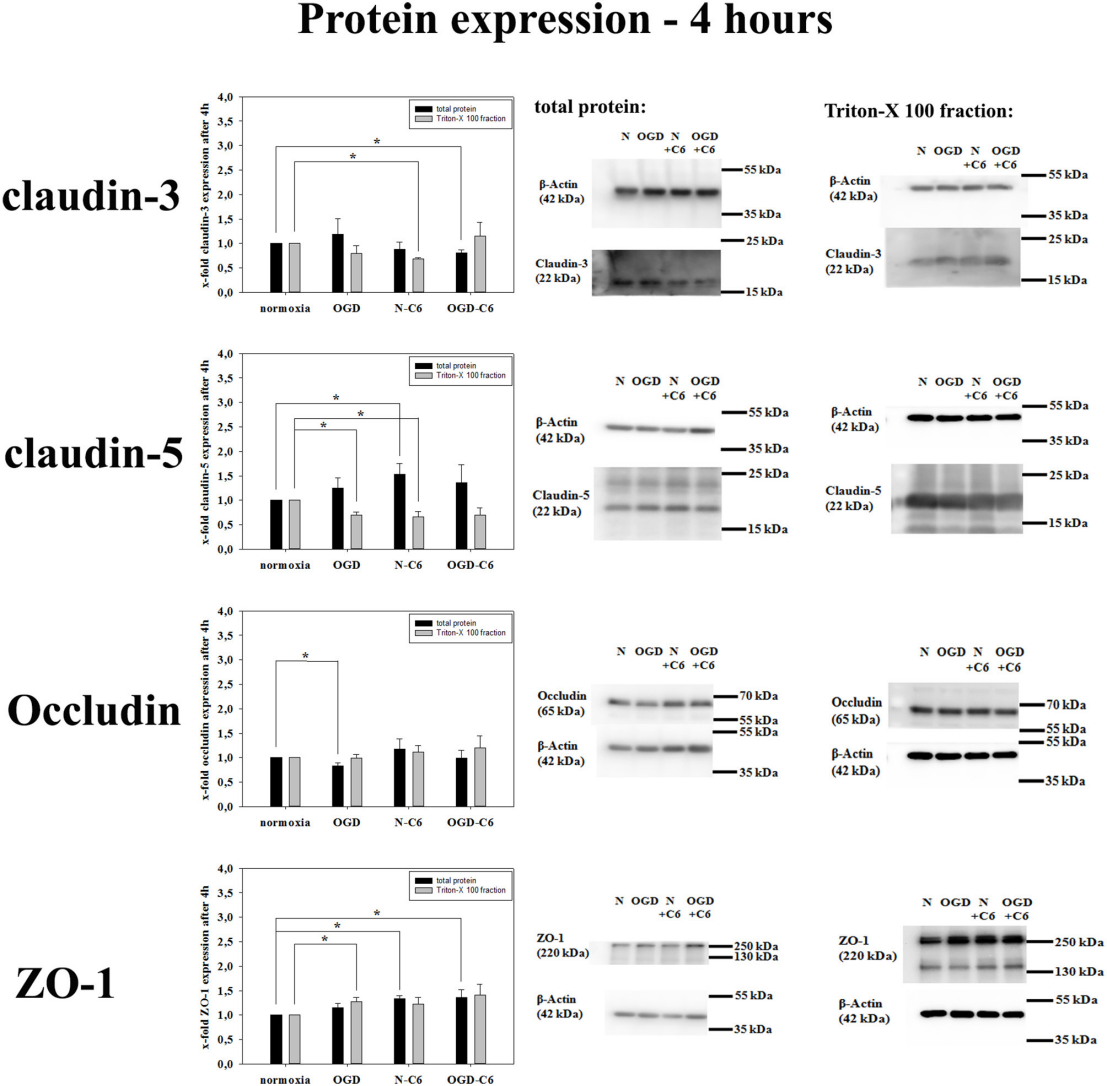
**Influence of 4 h oxygen/glucose deprivation (OGD) treatment and presence of astrocyte soluble factors on protein expression of tight junction proteins claudin-3, claudin-5, occluding, and ZO-1 of cerebEND cells**. On the left side results of densitometric analysis derived from western blots of total protein cell lysates as well as of Triton-X 100 membrane enriched fractions are presented, on the right side according representative western blot images are depicted. N, cerebEND normoxic control; OGD, cerebENDs treated under OGD conditions for 4 h; N−C6 or N+C6, cerebENDs treated for 4 h with C6 conditioned medium derived from C6 cells which were treated under normoxic control conditions for 4 h; OGD−C6 or OGD+C6, cerebENDs treated for 4 h OGD with C6 conditioned medium derived from C6 cells which were treated under OGD conditions for 4 h. Statistical significance was labeled with ^*^(*p* < 0.05, two-sided student's *t*-test with same variances). Data are presented as means ± s.e.m. (*n* = 4–6).

**Figure 3:**
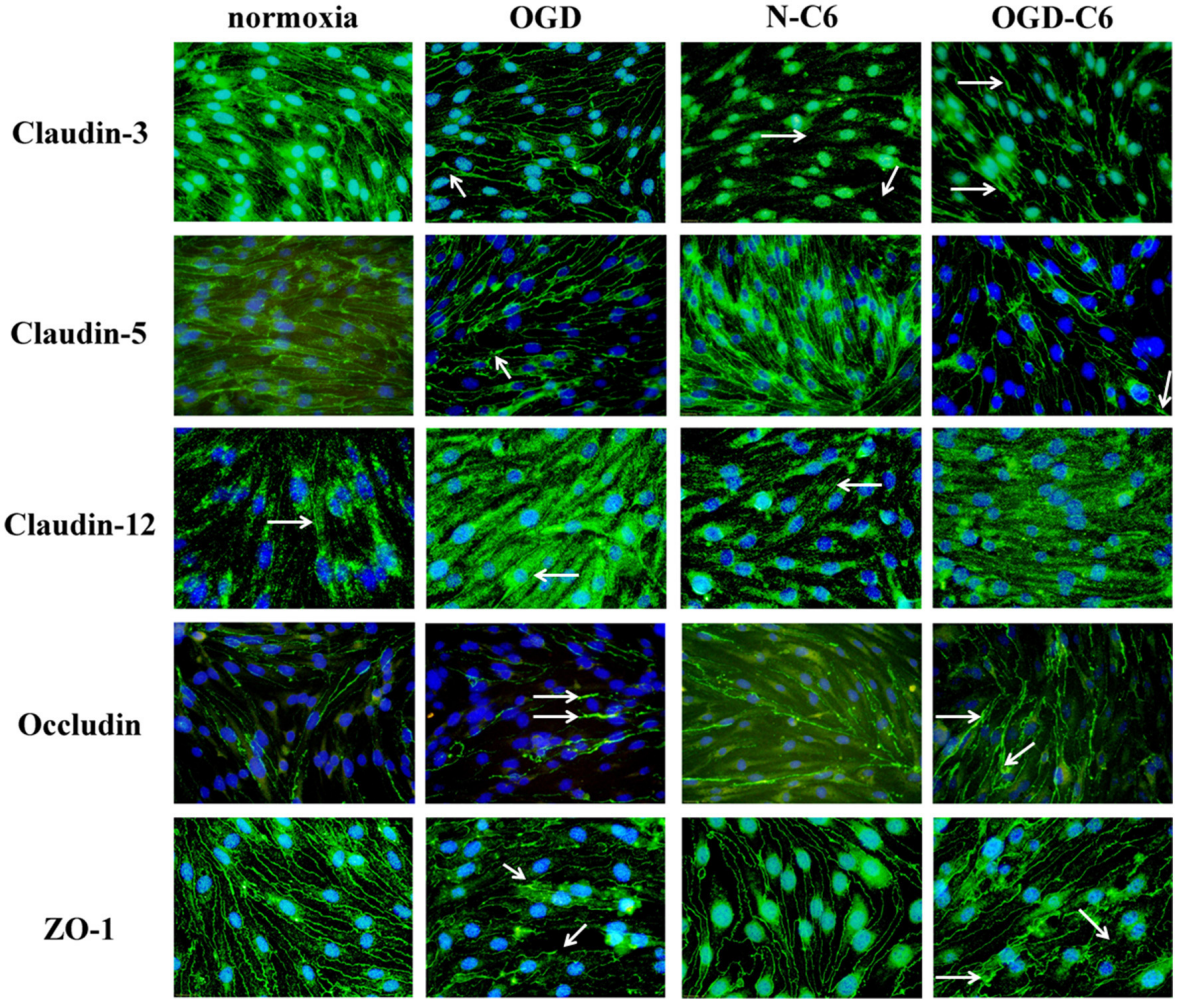
**Representative immunofluorescence images of tight junction proteins of cerebENDs treated for 4 h under normoxic or OGD conditions in the presence or absence of astrocyte soluble factors**. Normoxia, cerebEND normoxic control for 4 h; OGD, cerebENDs treated under OGD conditions for 4 h; N-C6, cerebENDs treated for 4 h with C6 conditioned medium derived from C6 cells which were treated under normoxic control conditions for 4 h; OGD-C6, cerebENDs treated for 4 h OGD with C6 conditioned medium derived from C6 cells which were treated under OGD conditions for 4 h. White arrows indicate lost tight junctions strands in case of claudin-3 N-C6, broadened tight junctions strands after OGD and OGD-C6 in case of claudin-3, claudin-5, and occludin, broadening and leaks in tight junction strands of ZO-1 OGD and OGD-C6 and non-continuous tight junction strands in case of claudin-12.

First, influence of astrocytes and OGD on mRNA levels of claudin-3, claudin-5, claudin-12, occludin and ZO-1 was measured by qPCR (Table 1). Claudin-3 was only downregulated by addition of astrocyte factors (73%, *p* < 0.05). In case of claudin-5, it was significantly downregulated by OGD and OGD-C6 to 65% and to 55% (*p* < 0.05), respectively, whereas mRNA expression of claudin-12 was not affected by any of the treatments. Similar to claudin-5, occludin was also significantly decreased by OGD and OGD-C6 to 38% and to 24%, respectively (*p* < 0.05). Finally, mRNA expression of ZO-1 was downregulated by OGD and OGD-C6 to 74% and to 64% (*p* < 0.05). In comparison to mRNA data western blots of total cell lysates revealed not such a significant regulation of tight junction protein expression (Figure 2). Claudin-3 was decreased by OGD-C6 to 81%, whereas claudin-5 protein expression was upregulated by N-C6 to 1.53-fold after 4 h treatment (*p* < 0.05). Claudin-12 was hardly detectable on western blots. Similar to mRNA regulation occludin was downregulated by OGD to 83% (*p* < 0.05), whereas ZO-1 was upregulated significantly by OGD and OGD-C6 to 1.34-fold and to 1.36-fold, respectively (*p* < 0.05). In contrast to western blots of total cell lysates, western blotting data with membrane enriched Triton-X 100 fractions were similar to mRNA expression data of claudins. In case of claudin-3, a reduction was only observed after N-C6 treatment (69%, *p* < 0.05). Claudin-5 expression was decreased after OGD, N-C6 and OGD-C6 to 70, 67, and 70%, respectively (*p* < 0.05). On the contrary, occludin expression of the 65 kDa band remained unchanged and ZO-1 also increased after OGD to 1.27-fold. Western blotting data proposed a change in localization of most tight junction proteins, which was confirmed by immunofluorescence microscopic images (Figure 3). Claudin-3 was significantly lost after treatment with astrocyte factors (N-C6), whereas it formed distinct continuous tight junction strands under other conditions. In case of claudin-12, no continuous tight junction strands surrounding the total cell were detected. OGD treatment seemed to cause broader tight junction strands of claudin-5, occludin and ZO-1, which could be due to a movement of tight junction proteins apart from the cell-cell contacts. Images of OGD-C6 treated cerebENDs showed a generally weaker staining density of tight junction strands in comparison to normoxia samples.

**Table 1:** **Influence of astrocytes and OGD on mRNA expression of tight junction molecules of cerebEND cells**.

	**Normoxia**	**OGD**	**N-C6**	**OGD-C6**
Claudin-3	1.00 ± 0.06	1.01 ± 0.11^§^	0.73 ± 0.05^*^^#^	0.82 ± 0.07
Claudin-5	1.00 ± 0.01	0.65 ± 0.08^*^^§^	1.04 ± 0.04^#^	0.55 ± 0.05^*^^§^
Claudin-12	1.00 ± 0.05	1.05 ± 0.05	1.02 ± 0.09	1.17 ± 0.09
Occludin	1.00 ± 0.02	0.38 ± 0.04^*^^§^	1.08 ± 0.11^#^	0.24 ± 0.02^*^^#§^
ZO-1	1.00 ± 0.05	0.74 ± 0.04^*^^§^	1.18 ± 0.11^#^	0.67 ± 0.07^*^^§^

Normoxia, cerebEND cells 4 h normoxia; OGD, cerebEND cells 4 h OGD; N-C6, cerebEND cells 4 h normoxia with C6-medium; OGD-C6, cerebEND cells 4 h OGD with C6-OGD medium. Data are presented as means ± s.e.m. (n = 8–10). Statistical significance was labeled with

* *vs. normoxia*,

# *significant vs. OGD*,

§ significant to N-C6 (p < 0.05, two-sided student's t-test with same variances).

### Influence of astrocytes and OGD on expression and functionality of Abc-transporters

**Figure 4:**
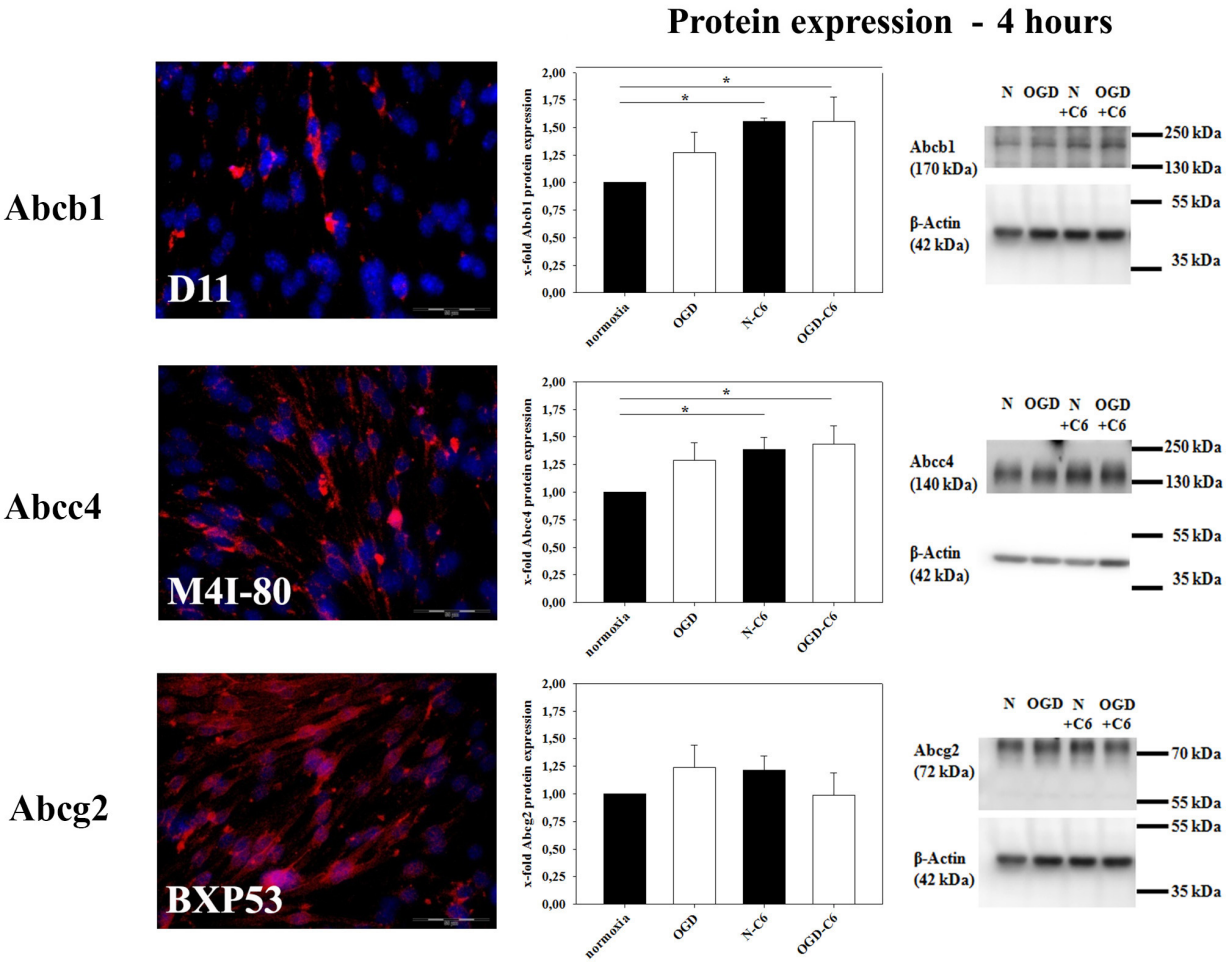
**Influence of 4 h oxygen/glucose deprivation (OGD) treatment and presence of astrocyte soluble factors on protein expression of Abc-transporters Abcb1, Abcc4, and Abcg2 of cerebEND cells**. Immunofluorescence images of cerebEND cells under normoxic conditions show the distribution of Abcb1, Abcc4, and Abcg2. In the middle, results of densitometric analysis derived from western blots of total protein cell lysates are presented, on the right side according representative western blot images are depicted. N, cerebEND normoxic control; OGD, cerebENDs treated under OGD conditions for 4 h; N−C6 or N+C6, cerebENDs treated for 4 h with C6 conditioned medium derived from C6 cells which were treated under normoxic control conditions for 4 h, OGD−C6 or OGD+C6, cerebENDs treated for 4 h OGD with C6 conditioned medium derived from C6 cells which were treated under OGD conditions for 4 h. Statistical significance was labeled with ^*^(*p* < 0.05, two-sided student's *t*-test with same variances). Data are presented as means ± s.e.m. (*n* = 4-6).

mRNA expression of Abcb1a was upregulated by astrocyte factors, OGD and OGD-C6 to 1.44-fold, 5.71-fold and 5.24-fold, respectively (*p* < 0.05) (Table 2). On the contrary, mRNA expression of Abcc4 was only signifcantly reduced by OGD-C6 treatment to 66% (*p* < 0.05). In case of Abcg2, mRNA expression was downregulated by OGD and OGD-C6 to 48 and 54%, respectively (*p* < 0.05). Western blotting of total cell lysates for Abcb1 confirmed the trend of mRNA data (Figure 4). Abcb1 protein was significantly upregulated by OGD (1.55-fold, *p* < 0.05) and OGD-C6 (1.56-fold, *p* < 0.05). In contrast to mRNA expression, Abcc4 protein was also upregulated by OGD (1.39-fold, *p* < 0.05) and OGD-C6 (1.44-fold, *p* < 0.05). In case of Abcg2 no significant regulation was found on the protein level after 4 h treatment. Immunofluorescence images confirmed that Abcg2 was the strongest expressed of the investigated Abc-transporters in cerebENDs, followed by Abcc4 and Abcb1 (Figure 4).

**Table 2:** **Influence of astrocytes and OGD on mRNA expression of Abc-transporters of cerebEND cells**.

	**Normoxia**	**OGD**	**N-C6**	**OGD-C6**
Abcb1a	1.00 ± 0.07	1.44 ± 0.17^*^^§^	5.71 ± 0.46^*^^#^	5.25 ± 0.72^*^^#^
Abcc4	1.00 ± 0.02	0.89 ± 0.15	1.07 ± 0.06	0.66 ± 0.02^*^^#^^§^
Abcg2	1.00 ± 0.03	0.48 ± 0.03^*^^§^	0.89 ± 0.06^#^	0.54 ± 0.05^*^^§^

Normoxia, cerebEND cells 4 h normoxia; OGD, cerebEND cells 4 h OGD; N-C6, cerebEND cells 4 h normoxia with C6-medium; OGD-C6; cerebEND cells 4 h OGD with C6-OGD medium. Data are presented as means ± s.e.m. (n = 8–10). Statistical significance was labeled with

* *vs. normoxia*,

# *significant vs. OGD*,

§ significant to N-C6 (p < 0.05, two-sided student's t-test with same variances).

**Figure 5:**
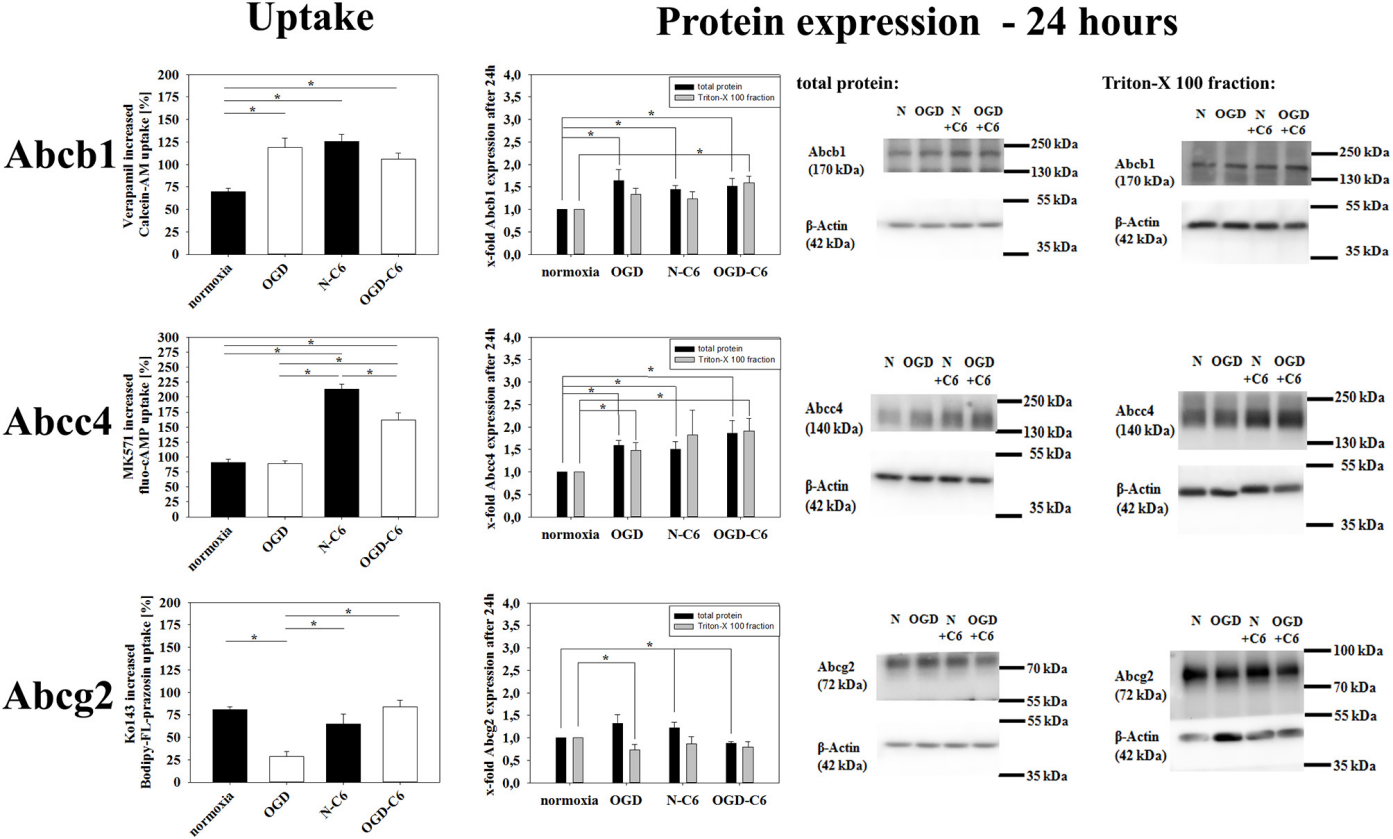
**Influence of 4 h oxygen/glucose deprivation (OGD) treatment with subsequent 20 h reoxygenation phase and presence of astrocyte soluble factors on Abc-transporter activity and protein expression**. On the left side results of uptake assays with specific substrates for Abcb1 (calcein-AM), Abcc4 (fluo-cAMP), and Abcg2 (Bodipy-FL-prazosin) are presented. In the middle densitometric analysis derived from western blots of total protein cell lysates as well as Triton-X 100 membrane enriched fractions are shown, on the right side according representative western blot images are depicted. N, cerebEND normoxic control; OGD, cerebENDs treated under OGD conditions for 4 + 20 h reoxygenation/renutrition; N−C6 or N+C6, cerebENDs treated for 24 h with C6 conditioned medium derived from C6 cells which were treated under normoxic control conditions for 4 h; OGD−C6 or OGD+C6, cerebENDs treated for 4 h OGD with C6 conditioned medium derived from C6 cells which were treated under OGD conditions for 4 h with a subsequent incubation of cerebENDs for 20 h with N−C6 medium. Statistical significance was labeled with ^*^(*p* < 0.05, two-sided student's *t*-test with same variances). Data are presented as means ± s.e.m. (*n* = 8–16 for uptake assays, *n* = 4–6 for protein expression data).

To test transporter functionality, uptake assays were performed for Abcb1, Abcc4, and Abcg2 with specific substrates (Calcein-AM, Bodipy-FL-Prazosin, fluo-cAMP). No significant effects on transporter functionalities were found after treatment for 4 h. Therefore, uptake assays were carried out after 24 h normoxia or 4 h OGD followed by 20 h normoxia. Verapamil increased the uptake of Abcb1 substrate calcein-AM by 69.53 ± 4.08% (*n* = 16) after normoxic treatment for 24 h (Figure 5). In concordance to upregulated Abcb1 expression, Abcb1 functionality was increased after OGD, N-C6 and OGD-C6 treatment resulting in a significant higher raise by verapamil by 118.92 ± 10.43% (OGD, *n* = 16), by 125.26 ± 8.36 (N-C6, *n* = 8) and by 105.81 ± 6.79% (OGD-C6, *n* = 8), respectively (*p* < 0.05). Uptake of Abcc4 substrate fluo-cAMP was increased by MK571 by 90.91 ± 5.14 (*p* < 0.05, *n* = 16) after 24 h normoxia. OGD treatment did not change the amount of increased uptaken fluo-cAMP by MK571 (89.21 ± 4.39, *n* = 16), whereas fluo-cAMP uptake differences by MK571 were elevated by N-C6 and by OGD-C6 by 213.38 ± 8.16% and by 161.59 ± 11.65%, respectively (*p* < 0.05, *n* = 8). In case of Abcg2, OGD reduced significantly the Ko143 increased uptake under normoxic conditions from 80.94 ± 2.71% (*n* = 16) to 28.31 ± 5.56% (*p* < 0.05, *n* = 16), whereas N-C6 and OGD-C6 treatments did not lead to significant changes. According to calcein-AM uptake data, western blotting showed upregulation of Abcb1 protein after 4 h OGD and subsequent 20 h normoxia, after 24 h N-C6 and 24 h OGD-C6 to 1.64-fold, to 1.45-fold and 1.52-fold (*p* < 0.05, *n* = 6), respectively. In case of Abcc4, OGD, N-C6 and OGD-C6 increased Abcc4 protein expression after 24 h to 1.59-fold (*p* < 0.05, *n* = 6), to 1.50-fold (*p* < 0.05, *n* = 6) and to 1.86-fold (*p* < 0.05, *n* = 6). In contrast to uptake data, Abgc2 western blots of total cell lysates revealed an increase after OGD (1.32-fold) and N-C6 (1.22-fold, *p* < 0.05), but interestingly a decrease after OGD-C6 (0.88-fold, *p* < 0.05). To elucidate the reason for the mismatching of functionality and Abcg2 protein expression data, western blots after 24 h treatment were repeated with membrane protein enriched fractions extracted with Triton-X 100. Protein expression data of Triton-X 100 fractions were similar to total protein lysates for Acbb1 and Abcc4 and corresponded well to functional uptake data (Abcb1: 1.52-fold OGD-C6; Abcc4: 1.48-fold OGD, 1.90-fold OGD-C6; *p* < 0.05, *n* = 6). In case of Abcg2, protein expression was significantly decreased by OGD to 0.73 ± 0.12-fold (*p* < 0.05, *n* = 4), whereas N-C6 nor OGD-C6 showed no significant differences in comparison to the normoxic control.

### Influence of astrocytes and OGD on expression and functionality of MMPs

**Figure 6:**
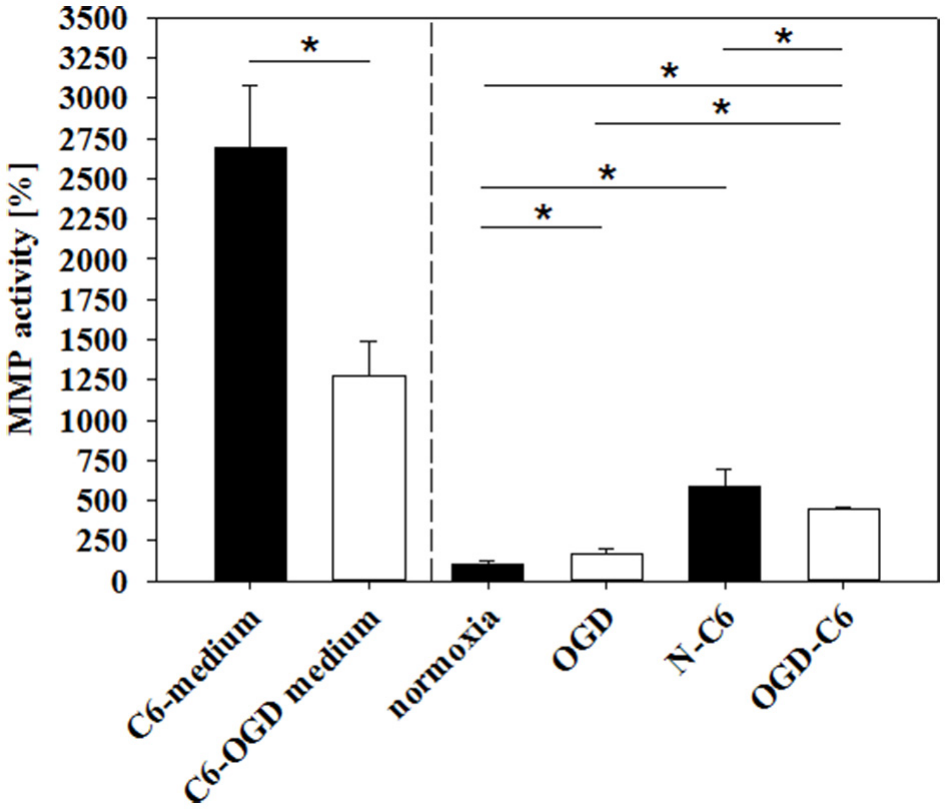
**Influence of 4 h oxygen/glucose deprivation (OGD) treatment and presence of astrocyte soluble factors on total MMP activity in medium supernatants**. C6-medium, supernatants of C6 cells after 4 h normoxia; C6-OGD medium, supernatants of C6 cells after 4 h OGD; normoxia, supernatants of cerebEND cells after 4 h normoxia; OGD, supernatants of cerebEND cells after 4 h OGD; N-C6, supernatants of cerebEND cells after 4 h normoxia treated with C6-medium; OGD-C6, supernatants of cerebEND cells after 4 h OGD treated with C6-OGD medium. Statistical significance was labeled with ^*^(*p* < 0.05, two-sided student's *t*-test with same variances). Data are presented as means ± s.e.m. (*n* = 8–19).

Matrixmetalloproteinases (MMPs) play a pivotal role in the degradation processes of basal lamina as well as of tight junction proteins at the blood-brain barrier during ischemic insults (reviewd in Jin et al., 2010). Therefore, changes of MMP2, MMP3, and MMP9 as well as from their inhibitors TIMP1 and TIMP3 of cerebENDs cells were investigated. Results were summarized in Table 3. Four hours of OGD significantly decreased TIMP-1 mRNA expression 88%, whereas increased TIMP-3 mRNA 1.39-fold (*p* < 0.05, *n* = 6). Addition of astrocyte factors (N-C6) increased MMP-3 expression 3.93-fold and TIMP-1 expression 1.47-fold, but decreased TIMP-3 expression to 79% (*p* < 0.05, *n* = 8). In case of OGD treatment with astrocyte factors (OGD-C6), MMP-2 expression was lowered to 92%, whereas TIMP-1 was upregulated to 1.39-fold (*p* < 0.05, *n* = 8). In order to test the functional relevance of these complex regulations, total enzyme activity of MMPs in medium supernatants was measured (Figure 6). Four hours OGD treatment of cerebEND cells significantly increased MMP activity to 164.31 ± 36.01% compared to normoxic control (100.00 ± 27.08%, *p* < 0.05, *n* = 8). Four hours of normoxic incubation of cerebENDs with astrocyte medium supernatants, which was incubated for 4 h under normoxic conditions before, led to an increase to 587.60 ± 105.47%, whereas addition of medium supernatants of OGD-treated astrocytes increased MMP-activity still significantly, but only to 446.07 ± 14.35% (*p* < 0.05). Analysing MMP-activity of C6 supernatants revealed a distinct higher activity for C6 supernatants in comparison to that obtained from cerebEND cells. Normoxic treated C6 cells secreted a MMP activity of 2694.62 ± 383.77% (*p* < 0.05, *n* = 14) in comparison to normoxic control of cerebENDs. OGD-treatment of C6 cells resulted in a significant decrease to 1271.67 ± 221.88% (*p* < 0.05, *n* = 12). Considering these MMP-activity start values for cerebEND incubation with C6 supernatants, a net increase of MMP-activity between N-C6 and OGD-C6 treatment of cerebEND cells of 60.86% could be calculated. In summary, OGD treatment increased MMP activity released by cerebENDs regardless of the presence of astrocytes.

**Table 3:** **Influence of astrocytes and OGD on mRNA expression of matrixmetalloproteinases MMP-2, -3, and -9, and TIMP-1 and TIMP-3 of cerebEND cells**.

	**Normoxia**	**OGD**	**N-C6**	**OGD-C6**
MMP-2	1.00 ± 0.02	0.95 ± 0.09	1.11 ± 0.06	0.92 ± 0.03^*^^§^
MMP-3	1.00 ± 0.03	0.88 ± 0.14	3.93 ± 1.07^*^^#^	3.12 ± 1.12
MMP-9	1.00 ± 0.02	0.95 ± 0.17	0.89 ± 0.19	1.10 ± 0.22
TIMP-1	1.00 ± 0.01	0.88 ± 0.04^*^^§^	1.47 ± 0.18^*^^#^	1.39 ± 0.15^*^^#^
TIMP-3	1.00 ± 0.05	1.39 ± 0.07^*^^§^	0.79 ± 0.04^*^^#^	1.02 ± 0.09^#^^§^

normoxia, cerebEND cells 4 h normoxia; OGD, cerebEND cells 4 h OGD; N-C6, cerebEND cells 4 h normoxia with C6-medium; OGD-C6, cerebEND cells 4 h OGD with C6-OGD medium. Data are presented as means ± s.e.m. (n = 6–8). Statistical significance was labeled with

* *vs. normoxia*,

# *significant vs. OGD*,

§ *significant to N-C6 (p < 0.05, two-sided student's t-test with same variances)*.

### Influence of astrocytes and OGD on expression and functionality of t-PA

**Figure 7:**
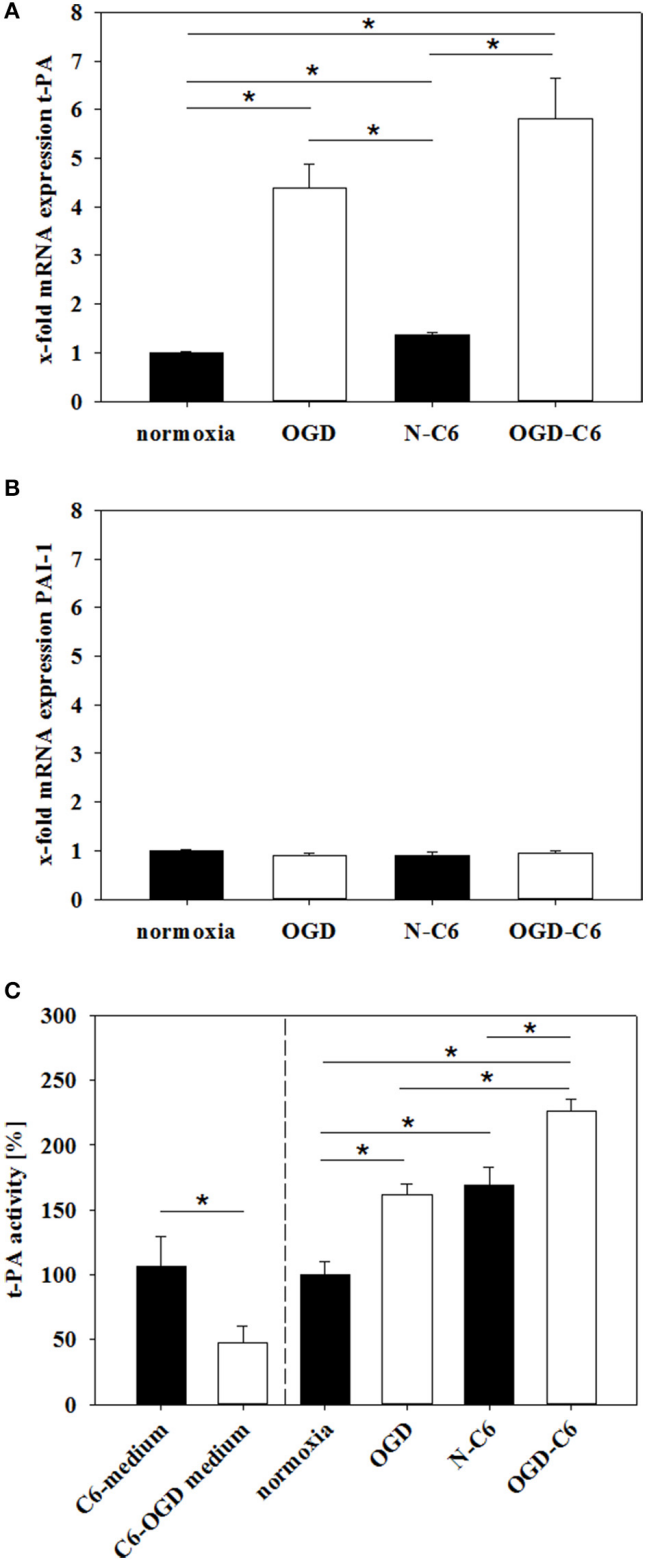
**Influence of 4 h oxygen/glucose deprivation (OGD) treatment and presence of astrocyte soluble factors on mRNA expression of t-PA (A) and PAI-1 (B) of cerebENDs and t-PA activity in medium supernatants (C)**. For mRNA expression data: N, cerebEND normoxic control; OGD, cerebENDs treated under OGD conditions for 4 h; N-C6, cerebENDs treated for 4 h with C6 conditioned medium derived from C6 cells which were treated under normoxic control conditions for 4 h, OGD-C6, cerebENDs treated for 4 h OGD with C6 conditioned medium derived from C6 cells which were treated under OGD conditions for 4 h; for t-PA activity data: C6-medium, supernatants of C6 cells after 4 h normoxia; C6-OGD medium, supernatants of C6 cells after 4 h OGD; normoxia, supernatants of cerebEND cells after 4 h normoxia; OGD, supernatants of cerebEND cells after 4 h OGD; N-C6, supernatants of cerebEND cells after 4 h normoxia treated with C6-medium; OGD-C6, supernatants of cerebEND cells after 4 h OGD treated with C6-OGD medium. Statistical significance was labeled with ^*^(*p* < 0.05, two-sided student's *t*-test with same variances). Data are presented as means ± s.e.m. (*n* = 6–8 for mRNA analysis, *n* = 3–13 for t-PA activity).

t-PA activity plays a major role in proteases cascades responsible for plasmin and MMP activation and following degradation of the basal lamina. Therefore, expression and functionality of t-PA and its inhibitor PAI-1 were assessed under normoxic and OGD conditions. Figure 7A shows an upregulation of t-PA mRNA expression in cerebEND cells by OGD treatment to 4.40-fold, by N-C6 to 1.36-fold and by OGD-C6 to 5.81-fold (*p* < 0.05, *n* = 6–8), whereas PAI-1 remained unregulated by any of the treatments (Figure 7B). To assess functional consequences of regulated t-PA, t-PA activity was measured in medium supernatants of cerebEND cells. Four hours of OGD significantly increased t-PA activity to 161.90 ± 8.17% in comparison to 100 ± 9.92% of the normoxia control (*p* < 0.05). N-C6 treatment raised t-PA activity to 169.42 ± 13.34%, which was further elevated to 226.66 ± 8.83% by OGD-C6 (*p* < 0.05). Interestingly, t-PA activity of medium supernatants derived from treated C6 cells decreased after OGD from 106.64 ± 22.61% to 47.11 ± 13.68% (*p* < 0.05, Figure 7C). These C6-medium supernatents were subsequently used to incubate cerebEND cells. Including the t-PA activity starting values of the C6-medium supernatants in the comparison of N-C6 vs. OGD-C6 data resulted in a real net t-PA activity increase of about 116% caused by cerebENDs.

### Influence of astrocytes and OGD on surface morphology of brain endothelial cells

**Figure 8:**
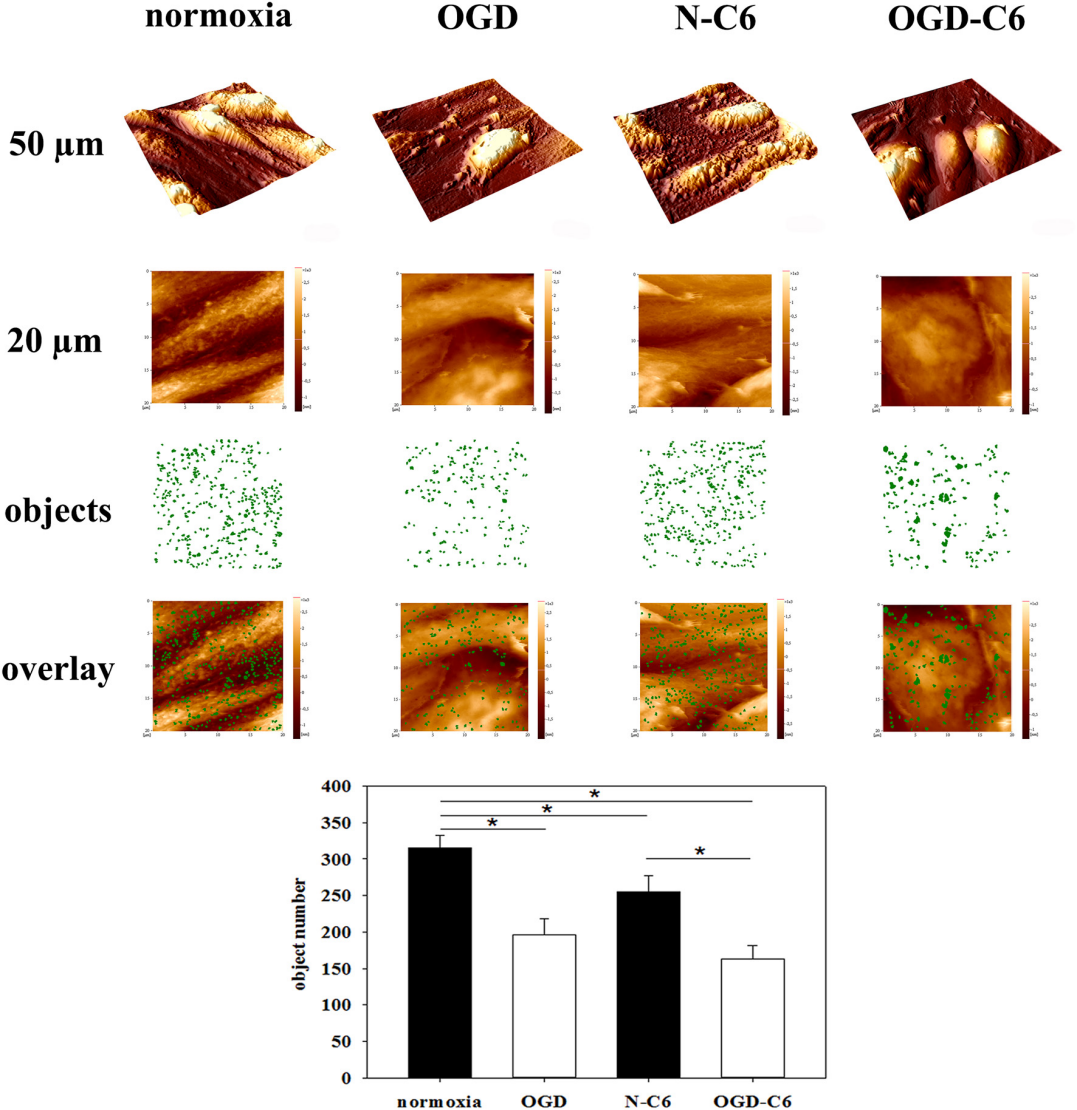
**Nanoscale surface topography analysis (nAnostic)**. CerebEND cells after 4 h oxygen/glucose-deprivation (OGD) and/or incubation with astrocyte conditioned medium were fixed by addition of glutardialdehyde (1% final conc.) and subjected to topography recording through atomic force microscopy (AFM) in buffer fluid—without any drying or labeling procedure. Quantitative analysis of protruding nano-objects is performed by computer vision. Shown are 3D-overviews of 50 μm (upper row), 20 μm raw data as taken for quantitative analysis (2nd row), a mask of identified nano-objects in green (3rd row) and the overlay of the two latter (lower row). Normoxia, cerebEND cells 4 h normoxia; OGD, cerebEND cells 4 h OGD; N-C6, cerebEND cells 4 h normoxia with C6-medium; OGD-C6, cerebEND cells 4 h OGD with C6-OGD medium. Statistical significance was indicated with an ^*^(*p* < 0.05 two-sided student's *t*-test with same variances). Data are presented as means ± s.e.m. of *n* = 20 images of (20 μm)^2^ area.

To evaluate whether different treatments changed the surface structure of cerebEND cells, their surface was analyzed at the nanoscale using atomic force microscopy (AFM). To objectively quantify the surface texture, a novel method was applied (nAnostic), which detects structures protruding from the mean surface level via computer vision. Interestingly, OGD treatment revealed a significant reduction of protruding nano-objects on the surface from 315 ± 18 to 196 ± 23 (*p* < 0.05, *n* = 20, Figure 8). Addition of astrocyte factors (N-C6) also reduced the number of identified nanostructures to 255 ± 22, which was further decreased by OGD-C6 to 163 ± 18 objects (*p* < 0.05).

## Discussion

Ischemic insults such as stroke or traumatic brain injury induce changes of several facets of the blood-brain barrier (BBB). This comprises opening of the paracellular sealing by disorganization and disruption of tight junctions which thereafter contributes to vasogenic edema formation and worsened stroke outcome. But also Abc-transporter expression and functionality are changed leading to altered defense and protection against xenobiotics (drugs) as well as endogenous substrates (hormones). In addition to edema formation, this causes distinct disturbances in brain homeostasis and consequently damage of e.g., astrocytes or neurons via penetrated blood-born proteins (albumin) or changed ion concentration. Moreover, pharmacokinetics of drugs are influenced by dysregulated Abc-transporter activities. Therefore, it is of immense importance to unravel the complex underlying mechanisms to understand the pathology and to be able to develop novel therapeutic strategies.

In this context, it is known that astrocytes play a pivotal role during stroke pathogenesis. In the non-pathological status astrocytes are crucial for the maintenance of BBB characteristics (Janzer and Raff, 1987; Neuhaus et al., 1991; Deli and Joó, 1996; Ronaldson and Davis, 2012). They are suggested to regulate BBB permeability, water and ion exchange (Ballabh et al., 2004; Abbott et al., 2006; Mathiisen et al., 2010). In case of ischemic insults, the response of astrocytes is multifaceted. Astrocyte end feet cover over 95% of the brain capillary surface on the brain side. The injury of astrocytes results in a compromised BBB. Their local loss leads to a disassembly of the tight junction network and decreased expression of tight junction proteins such as occludin, claudin-5 or ZO-1. This correlates directly to paracellular leakiness (Willis et al., 2004, 2013; Shin et al., 2013). In addition, ischemia injured/activated astrocytes secrete chemokines (MCP-1, Rantes), pro—and anti-inflammatory cytokines (IL-1α, IL-1β, TNFα, interferon-γ) and growth factors (VEGF, bFGF, TGF-β). These molecules are known to directly activate processes at the brain endothelium to disrupt the BBB, promote angiogenesis or regulate transporters at the BBB (Lau and Yu, 2001; Lee et al., 2007; Doyle et al., 2010; Strecker et al., 2011; Vangilder et al., 2011; Ronaldson and Davis, 2012). Although these relations are known, no comprehensive *in vitro* study exist to unravel the effects of astrocytes, OGD or their combination on brain endothelial cells. Consequently, we studied the influence of astrocytes and OGD on an established blood-brain barrier (BBB) *in vitro* model based on murine cerebEND cells (Silwedel and Förster, 2006). CerebENDs represent an adequate BBB cell culture model forming significant tight monolayers with high TEER values comparable to models based on mouse primary brain endothelial cells (Silwedel and Förster, 2006; Takeshita et al., 2014). Moreover, cerebEND cells were successfully used for OGD studies and responded to TNFα-stimulus with a rapid increase of paracellular permeability (Silwedel and Förster, 2006; Neuhaus et al., 2012a). In the presented study permeability coefficients for the paracellular marker fluorescein were between 0.4–0.9^*^10^−3^ cm/min, which was in a similar range of other paracellular markers with a similar molecular weight (sucrose, Lucifer yellow) used for OGD *in vitro* studies with primary brain endothelial cells (Mysiorek et al., 2009). This range of permeability for such integrity markers (below 1^*^10^−3^ cm/min) was reported as adequate for validated BBB *in vitro* models in terms of *in vitro*/*in vivo* correlations (Lundquist et al., 2002; Culot et al., 2008; Mysiorek et al., 2009).

As first important parameter we have investigated the functionality of the physical barrier by measuring TEER and fluorescein permeability directly after OGD treatments. In experimental models of focal cerebral ischemia it was shown that sucrose, a vascular marker with low molecular weight, was able to permeate into the ipsilateral parenchymal hemisphere after transient middle cerebral artery occlusion (tMCAO) (Pfefferkorn and Rosenberg, 2003). Moreover, 2 h after tMCAO leaks in the BBB were found even for bigger molecules such as Evans Blue-albumin complexes (>60,000 Da) indicating a rapid opening of the BBB after stroke (Jiao et al., 2011). In concordance to this, we were able to show a significant BBB breakdown after OGD treatment in our *in vitro* model. Moreover, loss of barrier functionality was significantly increased by the presence of astrocytes during the OGD-treatment. Our data confirmed a previous OGD-study with a BBB co-culture model consisting of primary mouse endothelial and rat glial cells in which the presence of glial cells was necessary to disrupt the barrier significantly (Brillault et al., 2002; Mysiorek et al., 2009). In this context, vascular endothelial growth factor (VEGF) could be one major factor for BBB breakdown in our model. It was shown that VEGF was significantly upregulated in astrocytes after OGD or hypoxia, and it was postulated that this is responsible for BBB damage (Redzic et al., 2013). With regard to our model, C6 cells secreted significant amounts of VEGF which was further upregulated via hypoxia (Boveri et al., 2005; Yeh et al., 2008). Under our experimental conditions OGD increased mRNA levels of VEGF in C6 cells approximately two-fold (data not shown). In this context, it should be noted that cerebEND layers co-cultured with C6 cells exhibited lower TEER values in comparison to the mono-culture set-up before OGD experiments (presumably due to high basal VEGF levels and MMP activity (see Figure 6) in C6 medium supernatants). This fact has to be considered for data interpretation of fluorescein experiments (Figure 1B). In comparison to normoxia controls, N-C6 (normoxia+C6 cells) treatment resulted in increased TEER, but also apparently contradictory in elevated fluorescein permeability. TEER was determined before and after the experiments, whereas fluorescein was only measured at the end of the tests. Hence, fluorescein data reflected the absolute tightness status after the incubation periods, whereas TEER data presented the relative changes between before and after the treatments. Consequently, fluorescein permeability of N-C6 (normoxia+C6 cells) was higher than normoxic control (Figure 1B), because basal tightness of co-cultured cerebENDs was lower in the beginning of the OGD-experiment in comparison to mono-cultured cerebENDs. However, comparison of normoxic and OGD treated co-cultured cerebEND cells (normoxia+C6 cells vs. OGD+C6) revealed a very significant increase of fluorescein permeability after OGD-treatment, confirmed corresponding TEER data and thus demonstrated the additionally deleterious effects of astrocytes on BBB breakdown *in vitro*.

Increased BBB permeability for paracellular markers (sucrose, dextrans, Evans Blue-albumin) was directly correlated with changed expression or localization of tight junction proteins such as claudin-5, occludin or ZO-1 *in vivo* (Witt et al., 2003; Jiao et al., 2011). Redistribution of tight junction molecules were detected even after 2 h of reperfusion in a tMCAO rat model (Jiao et al., 2011). In concordance to this, OGD decreased mRNA expression of claudin-5, occludin and ZO-1 in our model significantly. Interestingly, presence of astrocytes did not influence mRNA levels of these tight junction proteins. Moreover, protein expression of tight junction proteins in total cell lysates was not strongly reduced. This was in concordance to results of Engelhardt et al. (2014) who showed that total protein expression of claudin-5 (up to 48 h), occludin (up to 48 h) and ZO-1 (up to 8 h) were not decreased after several hours of hypoxia (1% O_2_) although TEER was reduced. Similar to our analysis, immunofluorescence images uncovered a delocalization of the investigated tight junction proteins. They and others reported that increased tyrosine-phosphorylation of tight junction molecules such as occludin or claudin-5 occurred which probably caused the disappearance of tight junction molecules from the cell boarders (András et al., 2007; Engelhardt et al., 2014). In this context, western blots of membrane enriched Triton-X 100 fractions confirmed the reduction of claudin-5 by OGD in our model and supported mRNA data as well as immunofluorescence images. Few data exist about claudin-3 and stroke. A recent study showed downregulated claudin-3 after 24 h after the surgery in a mouse model of intracerebral hemorrhage (Krafft et al., 2013). In our model expression of claudin-3 was not changed after 4 h of OGD treatment. Other claudins (claudin-1, claudin-12) are thought to be not significantly important for the barrier function in our model. Claudin-1 was hardly detectable by western blotting and was not found at cell-cell boarders on immunofluorescence images (data not shown). Claudin-12 formed non-continuous tight junction strands according to the postulation that claudin-12 itself can not form tight junctions (Piontek et al., 2011).

As next key property of the BBB, the expression and functionality of ABC-transporters were investigated. Few *in vivo* data are available about the regulation of ABC-transporters after stroke. Most of the reports showed an increase of expression as well as functionality of Abcb1 (Spudich et al., 2006; Lazarowski et al., 2007; Ueno et al., 2009). On the contrary, Dazert et al. (2006) found no differences in the regulation of Abcb1 on the mRNA level in his rat stroke model. In our model Abcb1 was significantly upregulated by C6 cells under normoxic conditions as well as OGD treatment. This was in concordance to other studies revealing that primary astrocytes as well as C6 cells were able to increase Abcb1 expression and functionality of brain endothelial cells (El Hafny et al., 1997; Gaillard et al., 2000; Berezowski et al., 2004). In case of Abcg2 few data exist about its regulation *in vivo* after stroke. For example, Dazert et al. (2006) published that Abcg2 was significantly upregulated at mRNA level after 14 days of reperfusion. In our model we were able to show for the first time that Abcg2 was downregulated by OGD in brain endothelial cells. In concordance to our *in vitro* data, own *in vivo* stroke studies revealed that Abcg2 protein expression of total brain samples was significantly decreased after 1 h tMCAO followed by 24 h reperfusion in male C57Bl/6 mice (unpublished data). Previous reports showed that inflammatory processes were mediated by pro-inflammatory cytokines such as TNFα within 2–6 h after the ischemic insult (Ronaldson and Davis, 2012). In this context and completely concordant to our expression as well as functionality data, it was shown that TNFα increased expression of ABCB1, but decreased expression of ABCG2 in a human BBB *in vitro* model (Poller et al., 2010). However, related to our *in vitro* data it can not be excluded that Abcg2 expression and functionality would be increased after a longer reperfusion phase after OGD as shown by Dazert et al. (2006).

Several studies reported that matrix metalloproteinases (MMPs) and tissue-type plasminogen activator (tPA) were involved in BBB disruption during stroke (Adibhatla and Hatcher, 2008; Bauer et al., 2010). As mentioned before, MMPs could degrade tight junction proteins claudin-5 and occludin as well as components of the basal lamina such as fibronectin, laminin and collagen leading to a disintegrated BBB and brain edema (Adibhatla and Hatcher, 2008; Cunningham et al., 2005; Yang and Rosenberg, 2011). Previously, MMP-2, MMP-3 and MMP-9 were shown to be the major MMPs in BBB disruption with a time-dependent activation and a very complex regulation (Jin et al., 2010). Moreover, MMP-9 levels were significantly increased after acute stroke in patients of a clinical study (Brouns et al., 2011). In our model, no significant regulation was found on the mRNA level of the investigated MMPs by OGD. However, according to the literature enzymatic activity of total MMPs of cerebEND cells was significantly upregulated by OGD in our model. This suggested that the MMP regulation took place mainly on the protein level. Moreover, recently Lenglet et al. (2014) proved the relevance of active MMP-1, MMP-10 and MMP-13 during stroke. These MMPs were not analyzed on the mRNA level in the presented study. Furthermore, protease t-PA can induce MMP-9 mediated BBB disruption and increase the MMP-1/TIMP-2, MMP-2/TIMP-2, MMP-8/TIMP-2 and MMP-9/TIMP-2 ratios in the hyperacute phase of reperfusion (Wang et al., 1998; Tsuji et al., 2005; Lenglet et al., 2014). In our model, expression as well as activity of t-PA of cerebEND cells was significantly upregulated by OGD according to the literature and can therefore also contribute to the observed barrier breakdown *in vitro*. Interestingly, in contrast to MMP activity, presence of astrocyte factors increased t-PA activity of cerebEND cells after OGD.

The novel Nanostic-AFM method enabled us to detect and quantify changes of brain endothelial cells after OGD treatments via a morphological approach even at the nanometer scale. Of note, the topographical alterations focus on local height distributions which are non-detectable for light microscopy. With the brain endothelial cells investigated here, OGD significantly reduced the number of microvilli-like objects. In epithelial cell layers the number of counted nanostructures (objects) on the cell surface was related to the cytoskeletal organization and the degree of cellular differentiation (Thoelking et al., 2010). In brain endothelial cells tight junction proteins are connected to the cytoskeleton (Abbott et al., 2006; Ronaldson and Davis, 2012). In our model, the link between the number of objects to an underlying cytoskeletal remodeling has to be shown in further studies. However, it could be hypothesized that a decreased number of nanostructures could be associated to the already shown redistribution of tight junction proteins and loss of barrier after OGD.

In summary, we were able to show that the influence of astrocytes in our BBB *in vitro* stroke model is of immense importance comprising the physical and the transport barrier as well as major BBB damaging proteases. Consequently, we would recommend to include astrocytes in BBB *in vitro* stroke models according to the concept of the neurovascular unit and its role during cerebral ischemia (Berezowski et al., 2012). However, it has to be pointed out that the brain endothelium *in vivo* is additionally regulated by blood flow and several other cell types of its microenvironment (pericytes, microglia, oligodendrocytes, blood cells). *In vivo* processes causing BBB disruption and repair during and after stroke take place in a complex regulatory network which is not possible to be simulated entirely in our model. Also, we can not exclude that results obtained from a BBB model with a 3D capillary architecture based on the same cells as used in this study would differ from the presented ones. Therefore, future studies with *in vivo* stroke models have to elucidate concordance and relevance of our *in vitro* findings.

### Conflict of interest statement

The authors declare that the research was conducted in the absence of any commercial or financial relationships that could be construed as a potential conflict of interest.
