# Supplementary material for: The pivotal role of astrocytes in an in vitro stroke model of the blood-brain barrier
Source: Front Cell Neurosci. 2014 Oct 28;8:352. doi: 10.3389/fncel.2014.00352 (PMC4211409; doi:10.3389/fncel.2014.00352)
Supplement: Supplementary file 1 [file Table1.PDF]

Table 1S: List of Taqman-probes used for qPCR

Target	Taqman® probe identification number
Abcb1a	Mm00440761_m1
Abcc4	Mm01226380_m1
Abcg2	Mm00496364_m1
Angiopoietin-2	Mm00545822_m1
β-actin	Mm01205647_g1
Caveolin-1	Mm00483057_m1
Claudin-1	Mm00516701_m1
Claudin-3	Mm00515499_s1
Claudin-5	Mm00727012_s1
Claudin-12	Mm01316511_m1
Hif1a	Mm00468869_m1
Kdr (VEGFR2)	Mm00440099_m1
Lrp1	Mm00464608_m1
MMP-2	Mm00439498_m1
MMP-3	Mm00440295_m1
MMP-9	Mm00442991_m1
Neuropilin-1	Mm00435379_m1
Occludin	Mm00500912_m1
Plat (t-PA)	Mm00476931_m1
Serpine1 (PAI-1)	Mm00435860_m1
Tek (Tie-2)	Mm00443243_m1
Timp1	Mm00441818_m1
Timp3	Mm00441826_m1
Tjp1 (ZO-1)	Mm01320637_m1
Vegfa	Mm01281449_m1
