# Supplementary material for: The pivotal role of astrocytes in an in vitro stroke model of the blood-brain barrier
Source: Front Cell Neurosci. 2014 Oct 28;8:352. doi: 10.3389/fncel.2014.00352 (PMC4211409; doi:10.3389/fncel.2014.00352)
Supplement: Supplementary file 2 [file Table2.PDF]

Table 2S: List of antibodies used for western blotting and immunofluorescence microscopy

Target	Product number, Company	Species	Application dilution
Abcb1	sc-55510 (D11), Santa Cruz<br>ALX-801-002 (C219), Enzo Life Sciences	rabbit<br>mouse	1:100 for IF<br>1:20 for WB
Abcc4	ALX-801-039(M4I-80), Enzo Life Sciences	rat	1:100 for IF and WB
Abcg2	ab24115 (BXP53), Abcam	rat	1:100 for IF and WB
β-actin	A3853, SigmaAldrich<br>A3854[$], SigmaAldrich	mouse<br>mouse	1:10,000 for WB<br>1:25,000 for WB
Claudin-1[*]	51-9000, Zymed®, Invitrogen	rabbit	1:50 (IF), 1:100 (WB)
Claudin-3	34-1700, Zymed®, Invitrogen	rabbit	1:100 for IF and WB
Claudin-5	34-1600, Zymed®, Invitrogen	rabbit	1:100 for IF, 1:200 for WB
Claudin-12	18801, IBL	rabbit	1:50 (IF), 1:100 (WB)
Occludin	71-5000<br>33-1500	Rabbit<br>mouse	1:400 for WB<br>1:200 for IF
VE-cadherin	Hybridoma supernatant of monoclonal antibody 11D4.1 #	rat	undiluted
ZO-1	40-2300, Zymed®, Invitrogen	rabbit	1:100 for IF and WB
HRP-anti-mouse	LNA931V/AG, GE Healthcare UK ltd.		1:5000 for WB
HRP anti-rabbit	LNA934V/AG, GE Healthcare UK ltd		1:5000 for WB
HRP anti-rabbit	12015218001, Roche Diagnostics		1:5000 for WB
HRP anti-rat	61-9520, Invitrogen		1:5000 for WB
anti-rat IgG Alexa Fluor 488	A21208, Invitrogen	donkey	1:200 for IF
anti-rabbit IgG Alexa Fluor 488	A21206, Invitrogen	donkey	1:200 for IF
anti-mouse IgG Alexa Fluor 488	A21202, Invitrogen	donkey	1:200 for IF

WB = western blotting, IF = immunofluorescence microscopy

*: used together with HRP-anti-rabbit secondary antibody from Roche Diagenostics (12015218001).

#: directed to the ectodomain of mouse VE-cadherin, see also Gotsch U, Borges E, Bosse R, Boggemeyer E, Simon M, Mossmann H, Vestweber D. 1997. VE-cadherin antibody accelerates neutrophil recruitment in vivo. J Cell Sci 110:583–588.

$: Peroxidase-linked antibody, usage of secondary antibodies was not applied
