# Supplementary material for: The pivotal role of astrocytes in an in vitro stroke model of the blood-brain barrier
Source: Front Cell Neurosci. 2014 Oct 28;8:352. doi: 10.3389/fncel.2014.00352 (PMC4211409; doi:10.3389/fncel.2014.00352)
Supplement: Supplementary file 3 [file Table3.PDF]

Table 3S: Influence of astrocytes and OGD on mRNA expression of angiopoietin-2 (Angpt2), caveolin-1 (Cav-1), Lrp-1, neuropilin-1 (Nrp-1) and Tie-2 in cerebEND cells, normoxia = cerebEND cells 4h normoxia, OGD = cerebEND cells 4h OGD, N-C6 = cerebEND cells 4h normoxia with C6-medium, OGD-C6 = cerebEND cells 4h OGD with C6-OGD medium. Data are presented as means ± SEM (n=5-8). Statistical significance was labeled with * versus normoxia, #: significant versus OGD, §: significant to N-C6 ($p<0.05$, two-sided student's t-test with same variances).

	**normoxia**	**OGD**	**N-C6**	**OGD-C6**
**Angpt-2**	1.00 ± 0.02	2.36 ± 0.41*§	0.90 ± 0.11#	1.72 ± 0.35§
**Cav-1**	1.00 ± 0.02	0.75 ± 0.05*	0.74 ± 0.05*	0.50 ± 0.04*#§
**Lrp-1**	1.00 ± 0.01	0.97 ± 0.06§	6.44 ± 1.27*#	3.81 ± 0.41*#
**Nrp-1**	1.00 ± 0.02	0.97 ± 0.11	0.80 ± 0.04*	0.69 ± 0.04*#
**Tie-2**	1.00 ± 0.01	0.74 ± 0.06*§	1.16 ± 0.06*#	0.63 ± 0.05*§
