# Supplementary material for: The pivotal role of astrocytes in an in vitro stroke model of the blood-brain barrier
Source: Front Cell Neurosci. 2014 Oct 28;8:352. doi: 10.3389/fncel.2014.00352 (PMC4211409; doi:10.3389/fncel.2014.00352)

Figure 1S: Cultivation scheme for Transwell® non-contact co-culture set-up with cerebEND and C6 cells for subsequent physical barrier functionality studies (TEER, fluorescein permeability).

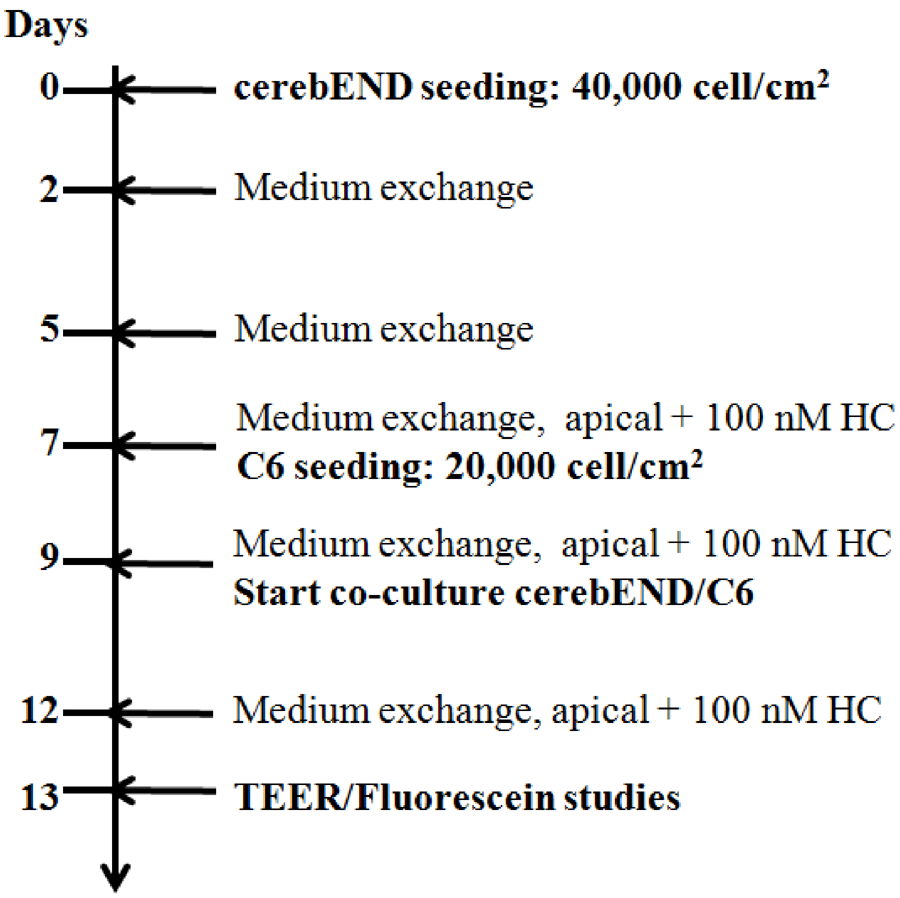

Supplement: Supplementary file 4 [file Image1.PDF]
