# Supplementary material for: The pivotal role of astrocytes in an in vitro stroke model of the blood-brain barrier
Source: Front Cell Neurosci. 2014 Oct 28;8:352. doi: 10.3389/fncel.2014.00352 (PMC4211409; doi:10.3389/fncel.2014.00352)

Figure 2S: Specificity of Abc-transporter substrates. Calcein-AM uptake was not increased by MK571 (general Abcc-blocker) or Ko143 (Abcg2-blocker) indicating that calcein-AM is a specific substrate for Abcb1 in the cerebEND model. Similar to that verapamil did not increase the uptake of Bodipy-FL-prazosin indicating that Bodipy-FL-prazosin is no Abcb1 substrate in cerebEND cells and thus can be defined as Abcg2-specific. Data are presented as means ± SEM (n= 8-24).

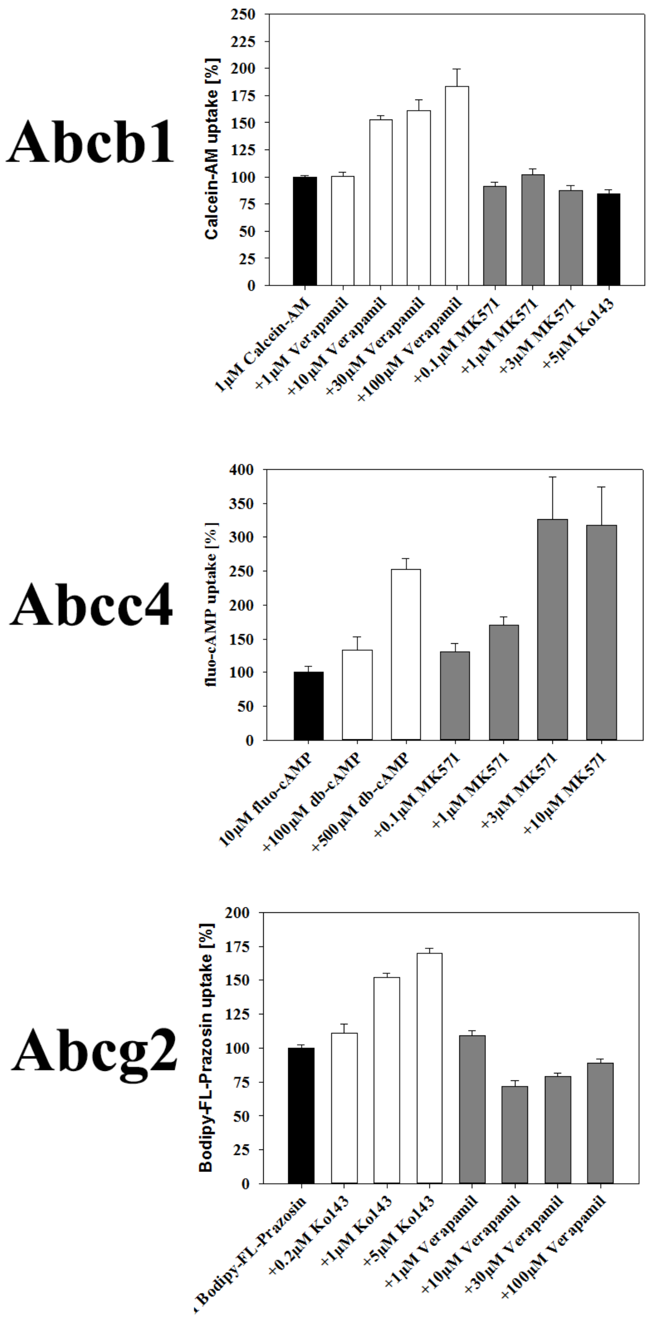

Supplement: Supplementary file 5 [file Image2.PDF]
