# Supplementary material for: The pivotal role of astrocytes in an in vitro stroke model of the blood-brain barrier
Source: Front Cell Neurosci. 2014 Oct 28;8:352. doi: 10.3389/fncel.2014.00352 (PMC4211409; doi:10.3389/fncel.2014.00352)

Figure 3S: Influence of 4h oxygen/glucose deprivation (OGD) treatment and presence of astrocyte soluble factors on mRNA expression of HIF-1a (A), VEGFa (B) and VEGFR2 (C) of cerebENDs. normoxia = cerebEND cells 4h normoxia, OGD = cerebEND cells 4h OGD, N-C6 = cerebEND cells 4h normoxia with C6-medium, OGD-C6 = cerebEND cells 4h OGD with C6-OGD medium. Statistical significance was labeled with * ($p<0.05$, two-sided student's t-test with same variances). Data are presented as means ± SEM (n= 6-8).

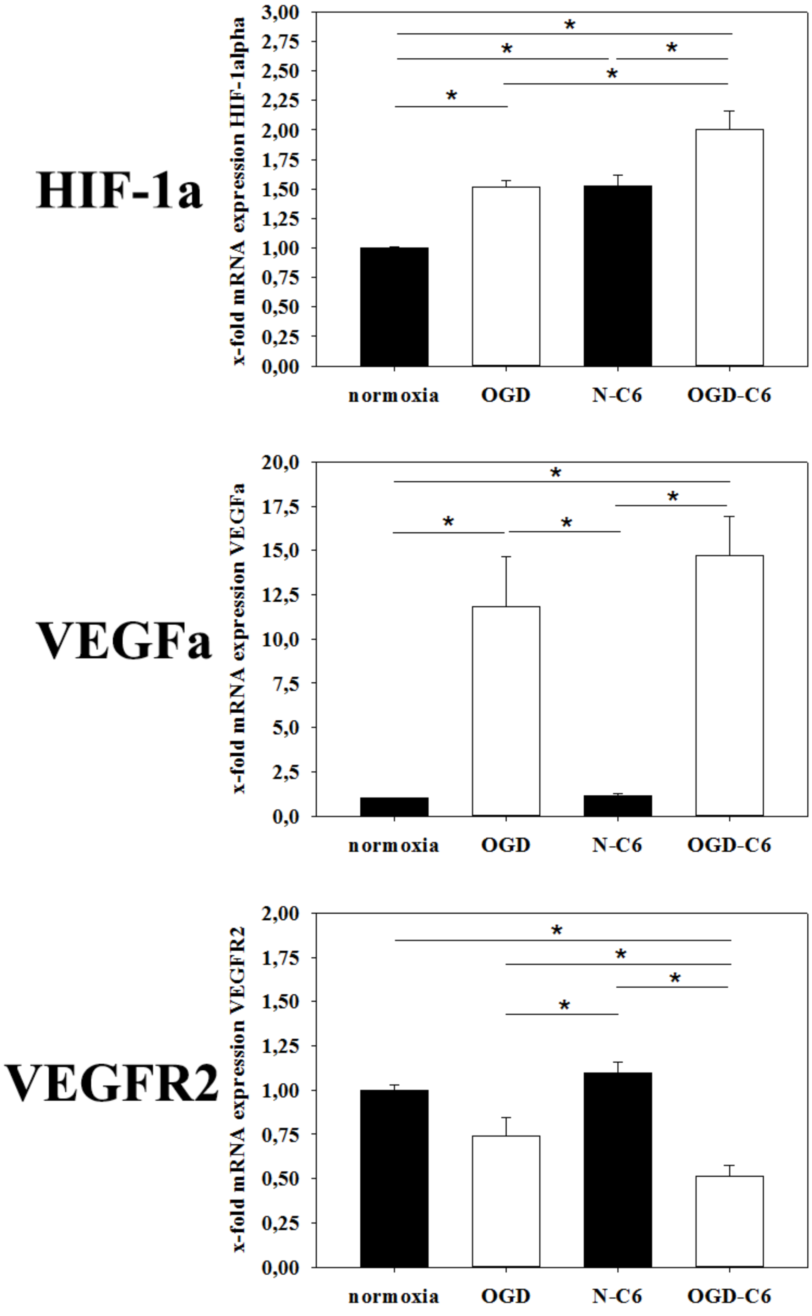

Supplement: Supplementary file 6 [file Image3.PDF]
